# Anticipation across modalities in children and adults: Relating anticipatory alpha rhythm lateralization, reaction time, and executive function

**DOI:** 10.1111/desc.13277

**Published:** 2022-06-07

**Authors:** Staci Meredith Weiss, Peter J. Marshall

**Affiliations:** ^1^ Department of Psychology Temple University Philadelphia Pennsylvania USA; ^2^ Department of Psychology University of Cambridge Cambridge UK

**Keywords:** alpha, anticipation, development, EEG, multimodal, prediction

## Abstract

The development of the ability to anticipate—as manifested by preparatory actions and neural activation related to the expectation of an upcoming stimulus—may play a key role in the ontogeny of cognitive skills more broadly. This preregistered study examined anticipatory brain potentials and behavioral responses (reaction time; RT) to anticipated target stimuli in relation to individual differences in the ability to use goals to direct action (as indexed by measures of executive function; EF). A cross‐sectional investigation was conducted in 40 adults (aged 18–25 years) and 40 children (aged 6–8 years) to examine the association of changes in the amplitude of modality‐specific alpha‐range rhythms in the electroencephalogram (EEG) during anticipation of lateralized visual, tactile, or auditory stimuli with inter‐ and intraindividual variation in RT and EF. Children and adults exhibited contralateral anticipatory reductions in the mu rhythm and the visual alpha rhythm for tactile and visual anticipation, respectively, indicating modality and spatially specific attention allocation. Variability in within‐subject anticipatory alpha lateralization (the difference between contralateral and ipsilateral alpha power) was related to single‐trial RT. This relation was more prominent in adults than in children, and was not apparent for auditory stimuli. Multilevel models indicated that interindividual differences in anticipatory mu rhythm lateralization contributed to the significant association with variability in EF, but this was not the case for visual or auditory alpha rhythms. Exploratory microstate analyses were undertaken to cluster global field power (GFP) into a distribution‐free temporal analysis examining developmental differences across samples and in relation to RT and EF. Anticipation is suggested as a developmental bridge construct connecting neuroscience, behavior, and cognition, with anticipatory EEG oscillations being discussed as quantifiable and potentially malleable indicators of stimulus prediction.

## Graphical abstract

Participants analyzed included 40 adults aged 18–20 and 40 children aged 6–8, who were cued with a sound that directed their attention to the location of an upcoming stimulus. The stimulus presented to the right or left of the participant in either the tactile, auditory or visual modalities and participants responded to the stimulus by pressing a foot pedal as quickly as possible. During this task, we recorded EEG brain activity and analyzed the power of a select band, alpha which oscillates from 8–12 Hz. Relative alpha power was calculated relative to baseline, averaged over the 500 ms prior to stimulus onset. Group averages for adults and children are displayed, with decreases in alpha power are indicated by blue, while increases are indicated in red. Adults show the most robust disengagement from the audiovisual cue, particularly at occipital sites and anticipatory decreases in alpha and mu power at contralateral central sites; anticipatory lateralization like this suggestions predictions are both specific to spatial location and sensory modality, especially when the expected stimulus was multi‐modal (visual or tactile). Children exhibit focal decreases in mu at contralateral central cites but exhibited less lateralization overall, such that their predictions were somewhat less spatially‐selective than adults, but still appear as modality‐specific. These differences in lateralization of alpha power per participant and trial were submitted for further analysis. Our multi‐level model examined variability between participants in executive function, as explained by each participant's reaction time and alpha power responses at the trial and individual level. Individual differences in executive function related to subject‐levelalpha lateralization, beyond trial‐level variability in reaction time. The magnitude of anticipatory mu was related to executive function but this differed in child and adults especially for anticipation of tactile stimuli, such that adults' α lateralization is positively associated with their executive function, while children's α lateralization is negatively associated with EF. This suggests adults and children might engage in different strategies for optimal prediction children who exhibit higher executive function might be more vigilant to unexpected stimuli, unlike adults with higher executive function, who seem to suppress processing at the uncued, unexpected spatial location.

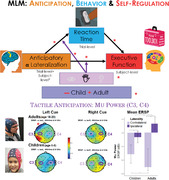

## 1 INTRODUCTION

Anticipation of the future perturbs and directs the flow of behavior: anticipation is evident and measurable in observable actions and quantifiable physiological activity (Ondobaka & Bekkering, 2012). The ability to anticipate an upcoming stimulus may be foundational to various domains of cognitive functioning, through refinement of possible avenues of behavior towards those consistent with executing goal‐oriented action (Vernon, 2014). It has been suggested that individual differences in anticipatory abilities evident early in life may be linked to later variability in cognitive outcomes, including language and executive function (EF) (Jaffe‐Dax et al., 2020; Reuter et al., 2018). However, there is little work focused on the significance of anticipation in relation to cognitive development during childhood, leaving a gap between classic behavioral work on future‐oriented processing in infants (Gomes et al., 2000; Haith, 1994; Hendry et al., 2016) and contemporary neural assessment of anticipatory in adults (Nobre & van Ede, 2018). The current study aims to fill this gap by examining individual differences in anticipatory neural responses in cross‐sectional samples of children aged 6–8 years and adults aged 18–25 years, using a novel adaptation of the classic Posner paradigm and a standard assessment of EF abilities.

A key charge of developmental science is to determine how component processes contribute to individual differences in cognitive abilities, both incrementally (across trials in a task) and developmentally (across ontogeny). Here we attempt to tease apart the functional role of anticipatory attention in accounting for trial‐by‐trial fluctuations in behavioral responses (indexed by reaction time; RT), as well as its relevance for individual differences in the ability to regulate behavior (indexed by performance on EF tasks). The current study builds on prior work with children that found an association between EF abilities and a developmentally salient neural measure of anticipation, desynchronization of the mu rhythm in the electroencephalogram (EEG), during anticipation of tactile stimulation (Weiss et al., 2018). In turn, this work complements studies linking children's cognitive abilities with indices of attentional orienting through the analysis of event‐related potential (ERP) responses to target stimuli during auditory (Isbell et al., 2016; Stevens et al., 2009) and visual (Shimi et al., 2014) selective attention tasks.

**RESEARCH HIGHLIGHTS**

- Prestimulus lateralization of visual alpha and sensorimotor mu rhythms was evident in anticipation of lateralized visual or tactile stimuli in children aged 6–8 years and adults aged 18–25 years.
- Single‐trial variability in prestimulus alpha lateralization was related to reaction time to visual and tactile stimuli, with a stronger association evident in adults than children.
- Individual differences in executive function were related to the extent of anticipatory lateralization of visual alpha and sensorimotor mu rhythms, controlling for the association of reaction time with trial‐level alpha lateralization.
- Significant anticipatory lateralization was evident for visual and sensorimotor alpha rhythms, but not for tau rhythm modulation in anticipation of an auditory stimulus.

The current investigation examines anticipatory alpha lateralization, a developmentally stable oscillatory marker of anticipation derived from the EEG in relation to inter‐ and intraindividual differences in EF. Prior studies have separately examined alpha lateralization relative to RT elicited by similar tasks in adults (Haegens et al., 2014; Kerr et al., 2011) and adolescents (Murphy et al., 2016). Here we examine developmental differences between children aged 6–8 years and young adults (18–25 years) in the role of stimulus anticipation as guided by proactive control, EF, and RT (Willoughby et al., 2018).

### 1.1 Neural indicators of anticipatory attention in children and adults

Within cognitive neuroscience, there has been sustained interest in the functional roles of brain oscillations as derived from the EEG (Buzsáki, 2004; Klimesch et al., 2007). Typical time‐frequency decomposition methods apply Gaussian‐curve sinusoidal signal processing methods to identify systematic changes in oscillatory responses. These oscillations are typically clustered into frequency bands that are believed to play distinct roles in neural communication (Fries, 2005; Lopes da Silva, 2013). Following preregistered hypotheses, the main focus of the current study was on the alpha rhythm (8–13 Hz in adults, lower in infants and children under 5 years of age), which plays distinct roles in coordinating action, perception, and cognition as well as in multisensory stimulus processing (Cravo et al., 2011; van Diepen & Mazaheri, 2017). Additional exploratory microstate analyses examined developmental differences across broader frequency ranges including beta (13–30 Hz) and theta (5–7 Hz) rhythms within the trials of the anticipatory cued attention task.

Paradigms that involve anticipation of visual stimuli have typically employed variations of the Posner paradigm, in which a central preparatory cue directs the participant's attention to one visual hemifield that will contain a target stimulus (Posner, 1980). Fluctuations in attentional states during such tasks can be tracked by examining the modulation of EEG oscillations that index rhythmic changes in the polarity of cortical tissue (Buzsáki, 2019). Such oscillations are thought to reflect the balance of postsynaptic potentials released by assemblies of pyramidal cells and inhibitory (GABAminergic) interneurons (Lopes da Silva, 2013), such that baseline synchronous activity is disrupted by the expectation of a stimulus (Foxe & Snyder, 2011). Allostatic changes in amplitude, phase, and frequency of oscillations evoked by expectation of an event can be computed using event‐related spectral perturbation (ERSP) methods, in which sinusoidal wavelets estimate the shift in the amplitude of each successive, overlapping time window (Makeig et al., 2004; Pfurtscheller et al., 1999).

As posited by the inhibition‐timing hypothesis (Foxe & Snyder, 2011; Klimesch et al., 2007), the presentation or the mere expectation of a stimulus invokes alpha rhythm desynchronization, or a reduction in power that can be termed an event‐related desynchronization (ERD). The deployment of attention to specific features of the environment disrupts the “default” baseline level of alpha synchronization, thus facilitating stimulus perception. In contrast, increases in alpha power can indicate both local (feature‐related) and global (state‐related) inhibition of attention (Arnal & Giraud, 2012). The time course of stimulus anticipation in neural activity accompanying spatially cued stimuli can be tracked via the lateralization of changes in alpha power, as characterized by the difference between contralateral desynchronization and ipsilateral synchronization (Haegens et al., 2012; Pfurtscheller et al., 1999). Patterns of anticipatory ERSP modulation involving spatially distinct combinations of changes are evident in visual, auditory, and tactile modalities across regionally specific alpha rhythms (Frey et al., 2015).

#### 1.1.1 Occipital alpha rhythm: Anticipation in the visual modality

It has been speculated that anticipatory alpha‐range EEG activity over posterior scalp regions (particularly occipital electrodes) in preparation for an upcoming visual stimulus is driven by a coordinated dorsal system that orients voluntary attention to the cued visual hemifield, while the ventral visual system monitors the uncued hemifield for unexpected stimuli (Capilla et al., 2014). This phenomenon is well‐characterized in adults, with lateralized visual alpha rhythm modulation evident over occipital sites following a cue to expect visual stimulation in the left or right hemifield (Worden et al., 2000). The extent of posterior alpha lateralization is associated with trial‐by‐trial visual discrimination performance and differences in this performance across individuals (Bengson et al., 2012; van Dijk et al., 2008). McKiney and Euler (2019) probed the association between higher‐order cognitive functions, RT, and lateralized alpha modulation using multilevel models separating within‐ and between‐subject variability in neural and behavioral measures. The authors employed a unimodal visual selective attention paradigm to identify an association of greater alpha modulation (though not explicitly contralateral or ipsilateral alpha) with greater fluid intelligence, with this effect being mediated by RT (McKiney & Euler, 2019).

Further, the functional role of oscillations in cognitive development and action regulation is evident in children older than 10 years, with increasingly closer coupling evident between RT and anticipatory neural modulation when comparing adolescents to adults (Murphy et al., 2016). However, relatively little literature has focused on the transition from early to middle childhood, when RT is dramatically more variable (Willoughby et al., 2018). Mento et al. (2018) studied beta modulation in relation to temporal orienting to a pair of visual stimuli in children aged 8–10 years: trials with greater beta desynchronization evident during the interstimulus period were associated with more rapid detection of the visual target stimulus. Further, the coupling of theta and beta was found to not only to predict target stimulus‐evoked RT, but reflected cue‐informed temporal expectancy. In children, a study of unimodal visual selective attention in 7– 10‐year‐olds (Vollebregt et al., 2015) found that the magnitude of posterior alpha‐range ERD during anticipation was not associated with RT to target stimuli, except when the spatial cue was invalid, in which case children with lower alpha amplitude exhibited greater RT. There remain open questions regarding how anticipatory alpha lateralization functions during multimodal cued paradigms, and if they explain differences in RT across development and stimulus sensory modality.

#### 1.1.2 Sensorimotor mu rhythm: Anticipation in the somatosensory modality

The sensorimotor mu rhythm has been implicated in the coupling of action and perception (Hari, 2006; Saby et al., 2013) and exhibits dissociable properties from the occipital alpha visual rhythm (Ritter et al., 2009; Yin et al., 2016). Mu oscillations are maximal over central electrodes over the postcentral gyrus, occurring in the 8–13 Hz range in adults and at lower frequencies (e.g., 6–9 Hz) in infants (Marshall et al., 2002; Thorpe et al., 2016). Haegens et al. (2012) reported that anticipatory mu desynchronization at central electrodes contralateral to the direction of a spatial cue was related to perceptual discrimination of (and RT to) tactile target stimuli (see also Anderson & Ding, 2011; Jones et al., 2010; Zhang & Ding, 2010). Adult studies have also found that the extent of mu synchronization (i.e., an increase in mu power) at ipsilateral central sites accounts for further variability in response time to target tactile stimuli (Gomez‐Ramirez et al., 2016; van Ede et al., 2014), action‐relevant cognitive skills (Weiss et al., 2020), and difficulties regulating attention in typical and atypical samples of children and adults (Chevalier et al., 2013).

#### 1.1.3 Temporoparietal tau rhythm: Anticipation in the auditory modality

The tau rhythm is an alpha‐range EEG oscillation over temporal and posterior parietal electrode sites that shows changes in amplitude (both in response and anticipation) of auditory stimulation (Karhson et al., 2015; Müller & Weisz, 2012). There is accumulating evidence that tau oscillatory activity may not lateralize in the same way as other alpha‐range rhythms. For example, bilateral poststimulus tau amplitude has been associated with verbal working memory in 10–14‐year‐olds MEG (Krause et al., 2010) and prestimulus bilateral changes in tau amplitude were observed in anticipation of speech sounds in adults (Mazaheri et al., 2014). However, one study found that the extent of lateralized tau modulation was associated with the *expected* salience of an upcoming stimulus, even when identical auditory stimuli were delivered (Hartmann et al., 2012).

#### 1.1.4 Multimodal anticipation: Individual differences in alpha modulation

One limitation of the existing visual work is that anticipation is difficult to isolate in a unimodal paradigm, where changes in alpha oscillations following presentation of a cue in a given modality and during anticipation of a target in the same modality occur over the same scalp region. Presenting a preparatory cue in a different modality from the target stimulus allows temporal and spatial differentiation of anticipatory activity from neural responses elicited by the cue (Foxe & Snyder, 2011; Mazaheri et al., 2014; Slagter et al., 2016).

Intraindividual variation in RT elicited by tactile and visual stimuli has been previously associated with the extent of anticipatory changes in adult sensorimotor mu and visual alpha (Katus et al., 2015). In adults, Gomez‐Ramirez et al. (2016) investigated the reactivity of sensory‐specific alpha oscillations to multisensory stimuli when cued to attend to one stimulus modality (auditory or visual) and inhibit attention in the other. Alpha ERD was observed at electrode sites over the cued sensory cortex, accompanied by alpha synchronization at ipsilateral electrode sites. Murphy et al. (2016) examined audio‐visual anticipatory alpha‐range modulation in small samples of children (8–12 years), adolescents (13–17 years), and adults. Across the entire sample, the ability to task‐switch was related to anticipatory alpha modulation, such that participants with more accurate stimulus detection exhibited on average greater contralateral suppression of anticipatory alpha power. Age‐related differences moderated this effect, with adolescents and adults exhibiting significant alpha ERD at occipital sites when a visual target was expected following a trial with an auditory target (compared to “repeat” trials that no required no switch from the previously cued modality), while this effect was not evident in children (Murphy et al., 2016).

Consistent with the behavioral literature, switching attention between modalities has been interpreted as more cognitively demanding for children than adults, reflecting gradual maturation of proactive cognitive control abilities (rather than reactive responses evoked by stimuli) and inhibition of the default or prior (Braver, 2012; Richardson et al., 2018). It is further speculated that anticipation may not develop at a consistent rate across all modalities, with particular developmental relevance for the primacy of somatosensory processing. Tactile stimuli are deployed in peripersonal space, embedded in action and sensorimotor feedback and in direct contact with the body (D'Souza et al., 2017), and therefore may engage attention in a different way relative to auditory or visual stimuli (Bremner et al., 2008; Macaluso & Maravita, 2010).

### 1.2 Alpha lateralization and the development and variability of action‐oriented cognition

Attentional control is increasingly understood as being related to prediction, which can be indexed through anticipatory neural responses and preparatory bodily actions (Braver, 2012; Clark, 2015; Engel et al., 2001). Classic neuroimaging work has revealed localized anticipatory biases in areas of the human visual cortex that facilitate subsequent processing of a target stimulus and involve cognitive mechanisms that are dissociated from reactive neural responses (Corbetta & Shulman, 2002; Kastner et al., 1999). These neural biases are not simply sustained, static signals, universal across trials or individuals; they appear to reflect individual differences in prior experience and personal stimulus relevance, further supporting claims of the brain as an organ that operates in an allostatic, prospective, and action‐oriented manner (Kok et al., 2012; Schulkin & Sterling, 2019). In generative and predictive processing accounts of cognition, prediction (vaguely or variably defined) serves as foundational to fluctuations of oscillatory brain activity and the regulation and speed of action, thus underlying the organization of behavior (Allen & Friston, 2018; Buzsáki, 2019; Clark, 1998).

In adults, RT to a target stimulus has been consistently associated with adult preparatory neural responses during an anticipatory epoch following a spatial cue to expect the target (Jensen & Mazaheri, 2010; Romei et al., 2010; Scheeringa et al., 2009). However, in children aged 6–10 years, prior work has not shown clear evidence of an association between anticipatory changes in sensory‐specific alpha rhythms and RT to target stimuli. Although sensory‐specific anticipatory alpha modulation is evident in children, as a function of greater alpha‐range synchronization at ipsilateral sites and/or reduced alpha‐range desynchronization at contralateral sites, more rapid RT was not associated with these values or alpha lateralization or modulation indices, which measure the difference of ipsilateral and contralateral change in alpha‐range amplitude, for visual (Vollebregt et al., 2015), auditory (Murphy et al., 2016), and tactile (Weiss et al., 2018) target stimuli.

Single‐trial RTs index intraindividual variability and individual differences in general speed of processing, which contribute to–but are distinct from–EF skills (Euler, 2018; Willoughby et al., 2018). Even for RT measures that do not exact demands on executive processes, there is a protracted period of development for the coordination of simple stimulus‐appropriate actions (Cepeda et al., 2013; Kail, 1991; Kail et al., 2016). Developmental improvements in RT can also be attributed to changes in myelination and the proportion of white to gray matter in the cortex (Scantlebury et al., 2014).

EF is understood as the constellation of cognitive skills involved in regulation of one's attention and actions in the pursuit of a goal. The planning, monitoring, and regulating of action‐based skills foundational to EF emerge gradually across infancy, becoming refined in early childhood (Garon et al., 2008; Gottwald et al., 2016; Nigg, 2017; Rueda et al., 2005). Measures of EF in the preschool years may have limited reliability and psychometric consistency (Wiebe et al., 2008) although by 6 years of age, EF scores stably predict later cognitive skills and academic performance (Blair & Razza, 2007; Bull & Scerif, 2001; Fuhs et al., 2014). The children in the current study range from 6 to 8 years of age, when consistent individual differences have become more stable and correspond with later variability in EF, but also at a point where profiles of selective attention and EF abilities are not yet adult‐like (Benitez et al., 2017). We hypothesized that there may be greater intraindividual differences across trials in children, whereas adults may have a more consistent neural response in anticipation of stimuli (Supplement 2, Preregistration).

It was expected that across sensory modalities, lower contralateral alpha band and heightened ipsilateral alpha band response during the cue‐stimulus interval will be associated with more rapid RTs in reporting a stimulus and better EF performance. In children, we expected lower contralateral band responses during the cue‐stimulus interval, but there is less evidence for lateralization driven by heightened inhibitory alpha during selective attention in younger participants. As a result, we expected significant differences in lateralization to be evident by participant age, with adults exhibiting greater differences in contralateral and ipsilateral alpha power compared to children. This may be further moderated by sensory modality, with the magnitude of alpha band lateralization most prominent in the tactile modality relative to the auditory and visual modalities.

The inhibition hypothesis of alpha‐range activity would be supported if we indeed observed a desynchronization associated with cognitive skills across all three modalities. Such a finding would lend support to “interactivity” accounts of supramodal spatial attention, suggesting that the local sensory‐specific alpha‐range modulations we expect to observe are dictated by a global attentional network. Further, we would then expect no significant differences in how identical auditory cues direct participants to monitor a corresponding bodily (peripersonal) location as opposed to a spatial (extrapersonal) location (Bremner et al., 2008). Another possibility is that there are differences among modalities; it is possible that occipital alpha modulation would account for more variance in EF, because the measures which index EF rely on visual discrimination as an outcome (Elke & Wiebe, 2017; Willoughby et al., 2018), thus indicating the centrality of visual representations in guiding anticipating of sensory events. We further suspect that if there are significant differences in the relations between EF across tau, mu, and occipital alpha rhythms, sensorimotor mu modulation might account for significantly more variance in EF. Candidate explanations for such a finding (which cannot easily be discerned by the proposed paradigm) would include (1) prominence and ease of eliciting of mu rhythm modulation, particularly in childhood; (2) centrality of the body in guiding action, particularly during anticipation; (3) novel/engaging nature of peripersonal tactile stimuli elicited greater variety in neural anticipation than unimodal auditory pairs of stimuli or the common audiovisual stimulus pairing.

Exploratory analyses of EEG microstates were also undertaken to elucidate whether the anticipatory lateralization evident was driven by interindividual variability in alpha (as suggested) and if these effects are restricted to the time period of anticipation selected. This allowed us to examine the spatially and temporal specificity of effects across frequency bands (theta/alpha/beta) in a data‐driven manner without being guided by band, spatial, or temporal‐specific hypotheses. Pioneering work in functional microstate analysis speculated that four prototypical functional states (labeled A–D) are involved in the intraindividual gradient of decreasing RTs over the course of development (Koenig et al., 2002), while a recent study has examined how anticipation of a visual stimulus induces microstate patterns akin to alpha desynchronization (Spadone et al., 2020). Previous studies have associated a specific microstate (B) with meditation and alertness accompanied by a lowering of physiological arousal (Spadone et al., 2020).

Microstate analysis tracks the changes of brain functional states, defined by specific distributions of simultaneously active brain regions, by identifying periods of stable scalp electrical potential topographies (Koenig et al., 2008). In adults, each time period of semistable global field power (GFP) is obtained from spatially defined microstate templates by clustering the distribution‐free data from all electrode sites into predetermined numbers of topographies or “microstate prototypes” (Michel & Koenig, 2018). The resulting metric, GFP is the standard deviation (SD) of the power at all electrodes of an average‐reference map, and serves as a useful index of individual variability in power across sites and frequency bands (Koenig et al., 2011).

Hierarchical clustering approaches are applied to data from all trials across participants, ensuring no (rather than minimal) overlap in microstates prototypes. Previous studies found that interindividual differences in alpha power largely determine intraindividual fluctuations in microstate at rest, and link them with distinct cognitive states (Croce et al., 2020; Milz et al., 2017).

The neuroscience literature on anticipatory alpha oscillations lends support for computational theories that embed prediction within the process of selective attention (Atzil & Barrett, 2017). This active field of inquiry in neuroscience (Nobre & van Ede, 2018; Shalev et al., 2019; Van Diepen et al., 2019) can be linked to developmental studies using behavioral measures (Garon et al., 2008; Veer et al., 2017) to test theories of prediction as central to cognition (Mento et al., 2020). In the current study, we picked up this thread to address key questions regarding the modal specificity of anticipatory lateralization and its functional coupling with action control across levels of measurement, individuals, and age group (children vs. adults).

### 1.3 The current study

This developmental investigation was undertaken to address a gap in the literature around the characterization of neural indices of anticipation in childhood and their relation to intra‐ and interindividual variability in control of action and goal‐directed behavior in children and adults. Patterns of anticipatory EEG lateralization were compared across three modalities (visual/tactile/auditory) and two age ranges (6–8 years and 18–25 years). This approach allowed us to test for age differences in the modality‐specific role of anticipatory EEG alpha‐range responses in relation to higher‐order cognitive functions. Our preregistered study (Supplement 2) assessed developmental differences in adult and child lateralized anticipatory alpha‐range EEG modulation by addressing three explicit research questions, using a combination of ANOVA and multilevel regression:

- Does the extent of anticipatory alpha‐range EEG modulation differ by sensory modality?
  Hypothesis A: Overall lateralization (contralateral minus ipsilateral alpha power) during anticipation will be most apparent in adults compared to children, particularly in the tactile and visual modality. Lateralization may not be readily evident in the unimodal auditory modality.
- Is the extent of anticipatory alpha‐range modulation related to behavioral indicators of attention or of stimulus perception (RT)?
  Hypothesis B: Anticipatory alpha modulation will be associated with EF scores (collapsed into a battery index combining flanker and card sort if results converge, tested across modality and age) within each age range.
- Are EF abilities related to the extent of alpha‐range EEG modulation in anticipation of auditory, tactile, or visual stimuli, beyond the inter‐ and intraindividual differences in RT?
  Hypothesis C: We expect overall individual differences in EF will hold their significance in relation to anticipatory alpha, remaining relevant despite accounting for inter‐ and intraindividual differences in RT. We further suggest that if there are significant differences in the relations between EF across tau, mu, and occipital alpha, sensorimotor mu modulation accounts for significantly more variance in EF due to the centrality of action control to the inhibition and flexibility evident implicated in both EF and individual development of mu variability.

The current study endeavored to examine whether the magnitude of anticipatory changes in alpha power was associated with indicators of goal‐directed action immediately relevant to the task paradigm: specifically, RT in response to target stimuli, further probing a gradual shift in the correspondence between age‐related changes in anticipatory alpha lateralization and stimulus RT that were evident when comparing older children to adolescents and adults (Murphy et al., 2016). A prior study found that the modulation of the mu rhythm following a visual cue that signaled which hand an upcoming tactile stimulus would be delivered to was associated with EF abilities in children aged 6–8 years (Weiss et al., 2018). One aim of the current study was to examine whether this relation with EF holds for anticipation in the visual and auditory modalities, or if anticipation of tactile stimuli elicits a stronger relation between individual differences in alpha (mu) modulation and EF, perhaps related to the salience of bodily action in developing the self‐regulation of action. Such a finding could also be a characteristic of the signal itself, with greater variability in the mu rhythm evident relative to tau and alpha rhythm, possibly due to the salience in the EEG signal and the consistency of mu oscillations within individuals.

Multilevel analyses further investigated whether alpha‐range activity during anticipation of auditory, visual, and tactile stimuli accounted for significant variance in EF abilities as measured using standardized scores on the Dimensional Change Card Sort (DCCS) and Flanker tasks from the NIH Cognitive Toolbox. Analyses employed a lateralization index (LI) as the difference between contralateral and ipsilateral ERSP in the alpha band (Haegens et al., 2012; McKiney & Euler, 2019; Murphy et al., 2016).

A series of hierarchical nested models parsed intraindividual and interindividual variability in EEG responses, testing (1) at the trial level, the association of RT and within‐subject lateralized ERSP (laterality index; LI), as moderated by modality and participant age group; (2) at the subject level, the association of EF and between‐subject LI, as moderated by modality and participant age group; (3) at the trial and subject level, the association of EF and between‐subject LI, as moderated by modality and participant age group. This preregistered analytic approach draws upon recent advances in statistical segregation of variability across single trials from participant‐specific variability in brain–behavior relations, an approach that has been successfully used with ERP and ERSP measures, as well as with RT and individual behavioral differences (Craddock et al., 2017; McKiney & Euler, 2019, Smith & Kutas, 2015).

Exploratory analyses further probed the difference in variance in GFP and its clustering across electrode sites into functional microstates, allowing identification of how the sequence, duration, and magnitude of EEG signal change within the course of a single trial, and examination of how the duration and power of these microstates vary across individuals in relation to other behavioral variables (RT and EF).

## 2 METHODS

### 2.1 Participants

In order to take part in the study, participants were required to have no medical or psychological diagnoses, be right‐handed, for English to be their native language (Albayay et al., 2019) and be free of long‐term medication. Forty‐nine adult undergraduate students consented to participate in return for course credit. After accounting for participant loss due to excessive artifacts (*n* = 2), technical problems (*n* = 2), and ineligibility (*n* = 5), the final sample of adults consisted of 40 participants aged 18–25 years (*M* = 20.91, *SD* = 4.22, 10 male). See the Supplemental Material for demographic characteristics of the samples, which included participants from a wide range of household backgrounds in both child and adult samples, and a significant proportion (47%) of participants from non‐white backgrounds.

We recruited 53 right‐handed children in order to retain a final sample of forty 6– 8‐year‐old children (*M* = 6.91 years, *SD* = 0.84, 23 male). Participant loss was due to various reasons including excessive artifact (*n* = 5), intolerance of the EEG cap (*n* = 3), technical problems (*n* = 4), and ineligibility (*n* = 1). Families who consented to participate received a $50 gift card for their time, and children received a small toy. An assent form was read to each child, who was provided with a custom comic book to color that narrated a superhero‐themed story reiterating the study protocol.

### 2.2 Task protocol

**FIGURE 1:**
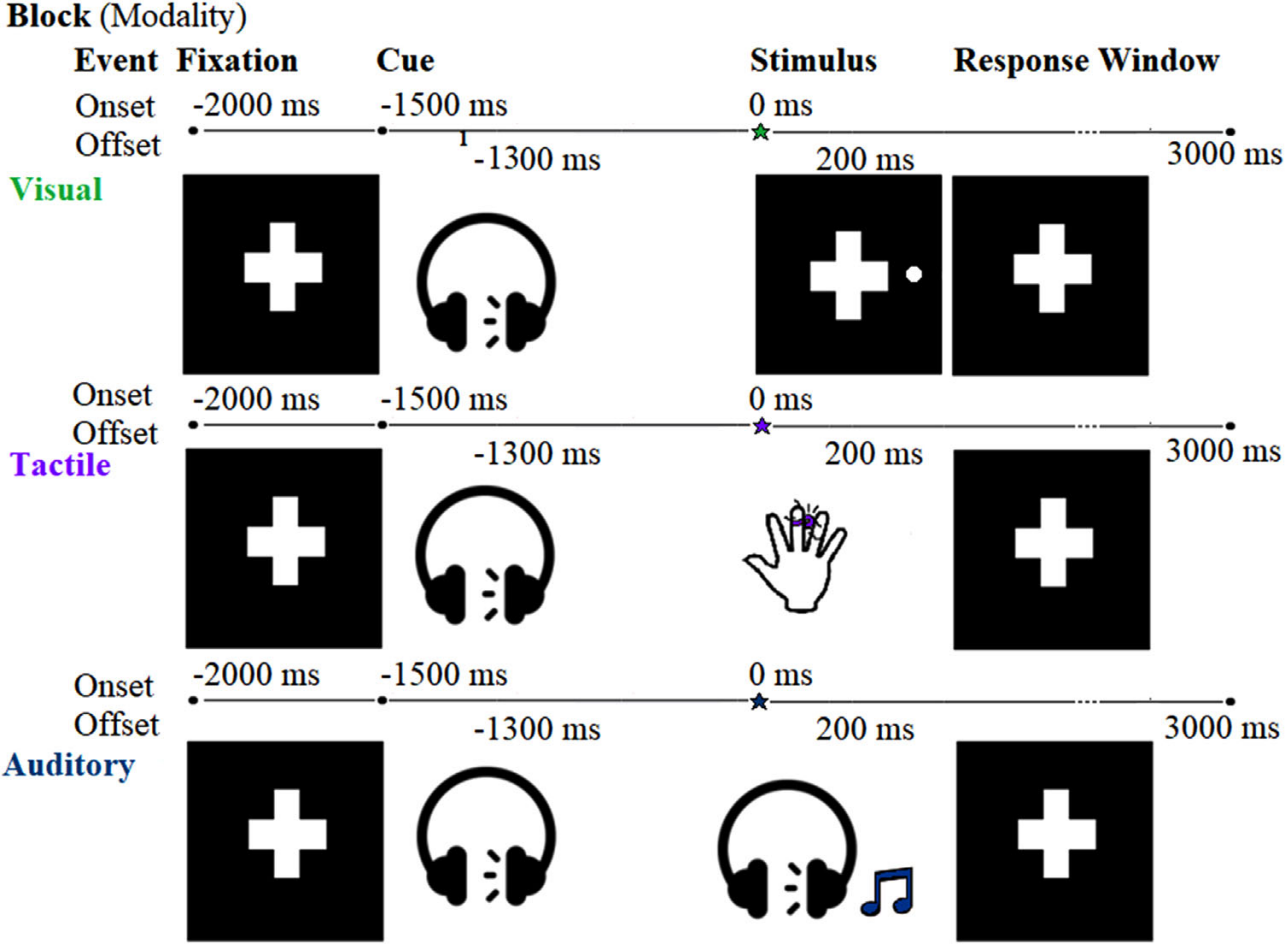
Schematic of the selective attention protocol. The multimodal selective spatial attention protocol was designed to elicit anticipation of visual, auditory, and tactile stimuli in three separate blocks, with the order of blocks counterbalanced across participants. A fixation point was displayed continuously for 2250 ms, with the onset of the low‐pitched auditory spatial cue (200 ms in duration) occurring 500 ms following the fixation onset. The onset of the target stimulus occurred 1500 ms later (at 0 ms, as indicated by the star). Participants were instructed to respond immediately after each target stimulus

The selective attention protocol for each trial involved a baseline fixation cross (+) displayed for 500 ms on a 17″ (43 cm) CRT monitor that was positioned around 1 m from the participant. In trials across all blocks, the fixation stimulus was followed by a spatially informative auditory cue, a low‐pitched (200 Hz) tone presented for 200 ms at 75 dB that was delivered to the left or right ear via insert earphones and that directed participants to attend to the left or right side of the monitor, their left or right hand, or their left or right ear (depending on the block) in anticipation of an upcoming target stimulus. The cue onset began a 1500‐ms prestimulus interval, after which the target stimulus was presented for 200 ms at the cued spatial location, with the sensory modality of the stimulation varying by block. Detailed parameters of the target stimuli can be found in the Supplemental Materials. The target stimulus consisted of visual probes in the cued field of vision, pulses of tactile stimuli to the cued hand, or target tones (600 Hz) to the cued ear (Figure 1). Participants were instructed to respond as quickly as possible to the target by pressing a foot pedal. An intertrial interval of 500 ms separated each trial, for a total trial duration of 4 s.

There were 360 trials in total, with 120 trials in each modality (presented in separate blocks, with opportunity for rest between blocks) and 60 trials to each spatial location (left/right) within each modality (auditory, tactile, or visual). Among these, 10 randomly interwoven trials consisted of “double” stimuli, in which an additional target stimulus in the attended modality was presented; participants were instructed to respond to these trials with two rapid foot pedal presses. The purpose of these trials was to ensure participants attended throughout the session. RT was measured as the time between target stimulus onset to the first‐foot pedal response.

### 2.3 EEG acquisition and processing

EEG signals were collected from 32 scalp sites using a stretch cap (ANT Neuro, Enschede, Netherlands) with Electro‐Gel conducting gel (Electro‐Cap, Eaton, OH). The recording sites were Fp1, Fp2, F3, F4, Fz, FC1, FC2, FCz, FC5, FC6, F7, F8, C3, C4, CP1, CP2, CPz, CP5, CP6, T7, T8, P3, P4, Pz, P7, P8, O1, O2, and the left and right mastoids (for additional EEG hardware and processing details, see Supplemental materials).

#### 2.3.1 EEG analyses

For each single‐target trial with a correct behavioral response, an epoch of 2750 ms was extracted beginning 2000 ms prior to onset of the target stimulus and extending 750 ms after target stimulus onset, including the behavioral response window (Delorme & Makeig, 2004). To keep a consistent number of trials across participants and groups, for each participant we selected 40 trials per condition to analyze with the least noise detected, selected from all usable trials shuffled randomly across the duration of the study, to prevent biasing from early versus later trials (Luck & Gaspelin, 2017).

**FIGURE 2:**
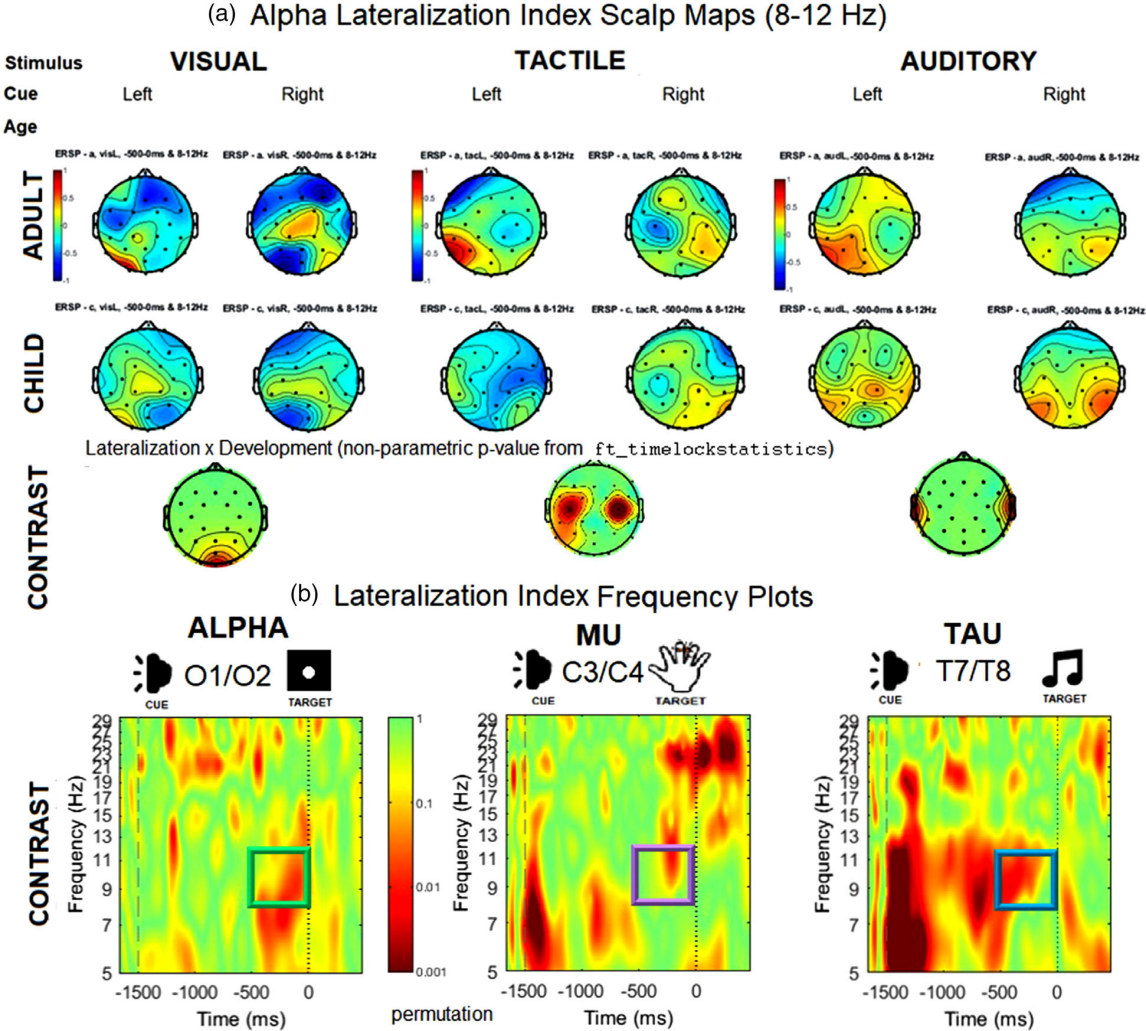
Scalp maps of mean anticipatory alpha ERSP and time‐frequency plots. Baseline‐corrected ERSP (in dB) is displayed at 32 electrode sites for the anticipatory period (from 500 ms prior to target stimulus onset at 0 ms), set to a consistent ERSP scale across cue direction, modality and group (−1 to 1 dB).

**FIGURE 3:**
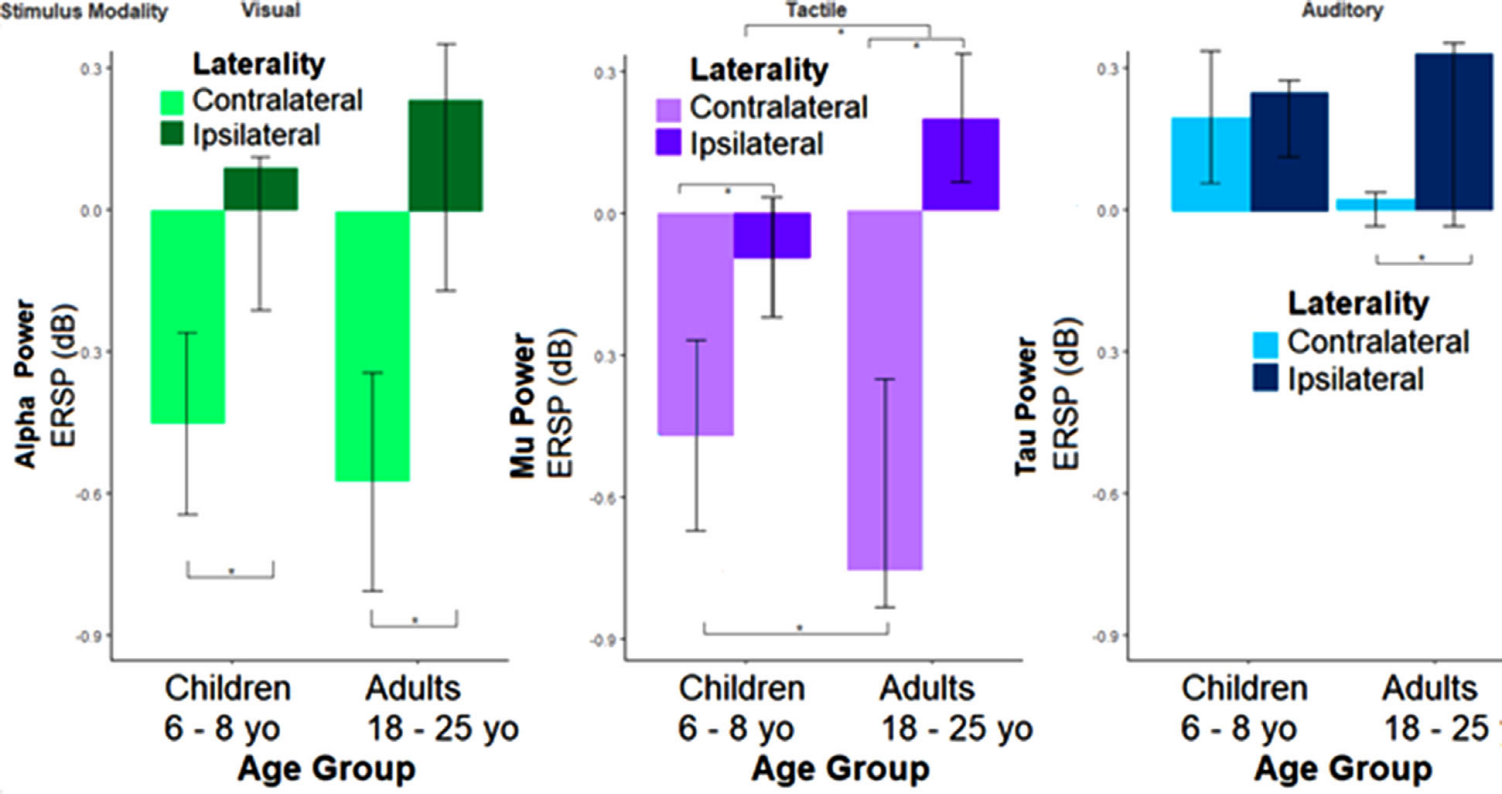
Bar charts of alpha ERSP by modality, age group, and hemisphere. Mean alpha‐range (8–13 Hz) ERSP and confidence interval of significance are displayed for electrodes of maximal contrast for each sensory stimulus, collapsed by laterality. As shown in Table 2, no significant asymmetries in response by cue direction were detected.

The envelope of the amplitude‐modulated signal was computed via the Hilbert transform (“hilbert” function in Matlab), which discards phase information and reveals fluctuations in oscillatory power over time. Corrections were also applied to the raw ERSP in order to control for fluctuations in 1/*f* aperiodic signal. Following a typical plan of analyses for lateralized alpha responses (Haegens et al., 2014, 2012), we identified electrodes of interest using a mass univariate approach that involved extracting the mean ERSP across all 32 sites and used a Bonferroni‐corrected permutated ranking to nominate the electrode sites with the greatest differences between ERSP at ipsilateral and contralateral sites by cue direction (left/right) and age group (child/adult) separately for each target stimulus modality (visual/tactile/auditory). These generated nonparametric contrast scalp maps, which established that the greatest contrasts were present between O1 and O2 for the visual stimuli, between C3 and C4 for the tactile stimuli, and between P7 and P8 for the auditory stimuli. These sites were further confirmed by examining the alpha distribution on scalp maps (Figure 2). Mean ERSP at each of these six electrode sites was extracted accordingly for further analyses (see Figure 3 for means according to modality, laterality, and group).

Using the results of the mass univariate scalp maps as a guide, a key variable submitted to ANOVAs and regressions was the mean alpha‐range ERSP (per trial or per subject) for the period from −500 to 0 ms where 0 ms is the onset of the target stimulus. Consistent with prior studies, this time window was selected to prevent contamination of anticipatory responses by changes evoked by the auditory cue that occurred at −1500 ms.

Once it was established that there were no significant differences by directional cue, for certain analyses, we collapsed mean alpha‐range ERSP into a LI subtracting contralateral ERSP from ipsilateral ERSP (Murphy et al., 2016). The following factors were entered to predict individual differences in ERSP values: direction of cue (left/right), sensory modality (visual/tactile/auditory), and participant age (child/adult).

### 2.4 Executive function battery

Following EEG collection, the cap was removed and participants carried out two EF tasks from the NIH Cognitive Assessment toolbox (see the Supplemental Information for background and specific task parameters). This computerized assessment includes a battery of tasks designed to parse the components of EF. Here we employed tasks designed to index cognitive flexibility (DCCS task) and inhibitory control (Flanker task). Age‐adjusted *t*‐standardized test scores were used (0–100 range, *M* = 50) as derived by the NIH Toolbox.

Participant scores on the Card Sort and Flanker tasks were calculated to reflect both accuracy and RT for participants who correctly identified targets on 80% of trials; accuracy alone is considered for participants who did not meet this threshold. Following NIH toolbox advice for measurement of EF (Zelazo et al., 2013) and existing procedures for group comparison mixed‐models, the age‐standardized scores from the Card Sort and Flanker tasks for each individual adult and child were averaged and group‐centered to form a sample‐standardized composite EF score.

### 2.5 Statistical analyses

Descriptive statistics for study variables are reported in Table 1, and demographics of the adult and child samples are reported in detail in the Supplementary materials. For all ANOVAs, within‐subject effects were adjusted using Greenhouse–Geisser correction factors; pairwise *t*‐test comparisons and multiple linear regressions are reported with *p*‐values adjusted for multiple comparisons using the FDR correction. Three 3‐way ANOVAs were conducted separately for each sensory modality, to investigate individual subject‐level differences in behavioral responses (RT) and participant‐mean ERSP amplitude during the anticipatory period. The following factors were entered to predict these outcomes: direction of cue (left/right), hemisphere (right/cue), and participant age (child/adult).

**TABLE 1:** Descriptive statistics for behavioral measures

	Children				Adults			
	Mean	SD	Skew	Kurtosis	Mean	SD	Skew	Kurtosis
Reaction time (ms)	357.33	0.48	−0.81	0.0	365.10	0.90	−0.69	0.05
Flanker	44.85	10.13	−0.42	0.57	49.52	9.30	−0.53	0.56
Card sort	51.46	13.10	−0.15	0.74	47.48	10.16	0.12	0.61
Language	50.85	1.22	−0.17	0.59	52.04	11.67	−0.29	0.70

To account for variability in single‐trial alpha power (within‐subjects) and individual differences in alpha power (between‐subjects), EF and RT were modeled using generalized linear mixed‐effects models (GLMMs). GLMMs allow for the inclusion of both random and fixed effects. Standard linear regression assumes independence of observations and is thus unsuitable for the analysis of a repeated‐measures design. GLMMs can incorporate the dependence structure of the observations into the model using random effects.

Multilevel models involved regressing EF (averaging the standardized age‐adjusted scores) over anticipatory alpha LI (calculated as contralateral–ipsilateral) at the prespecified, regionally specific electrode sites and its moderation by sensory modality (within‐subjects) and age group (between‐subjects), controlling for within‐subject variability in ERSP and RT. Post hoc regressions probed the association of EF with anticipatory ERSP by sensory modality (auditory, visual, or tactile), separately for ipsilateral or contralateral alpha power and participant age (child or adult). Post hoc tests are reported with *p*‐values adjusted for multiple comparisons using the Bonferroni correction.

To assess differences across participants in microstate spatial‐temporal functional clusters, the resulting GFP and duration (ms) of each microstate were submitted as the outcome in a linear mixed model analysis. GFP and duration of microstates were regressed on target stimulus modality (visual/tactile/auditory), developmental group (child/adult), and frequency band (theta/alpha/beta). Given the data‐driven nature and large number of data points reduced, Bonferroni‐corrected *p*‐values were employed for these analyses.

Outlier detection, defined as values with a Mahalanobi's distance of greater than 2.5, was performed on single‐trial and mean mu ERSP separately within child and adult samples, but no data points reached this threshold (Hadi, 1992). Normality assumption checks determined that the dependent variables in the analyses, mean mu ERSP, microstate GFP, and EF, were normally distributed. As expected, the distribution of RT as well as microstate duration was positively skewed. Transforming the RT variable did not result in a sufficiently unskewed distribution, and thus the multilevel models employed a Gamma distribution for RT rather than a Gaussian distribution. This approach considered the unimodal skewed distribution of RT, appropriate for variables with continuous values greater than 0 and the variance of values increasing proportionally to the mean (Lo & Andrews, 2015). Microstate duration was log‐transformed and submitted for mixed linear regression. Prior studies have successfully employed multilevel GLMMs to examine similar outcomes using EEG data at both the trial and individual level (Euler & McKinney, 2018; Craddock et al., 2017), but no study has examined developmental differences with mixed models. Our model was specified in R (R Core Team, 2016) using the lme4 package for model fitting (Bates & DebRoy, 2004; Craddock et al., 2017). Participant was specified as a random effect, and random slopes were estimated for all main effects and interactions. Likelihood ratio tests compared the goodness of fit of compatible models, which were performed by systematically removing each fixed effect term from the full model and comparing the log‐likelihood of the model with the term to the log‐likelihood of the model without the term. This provided a chi‐square statistic and a *p*‐value that indicated whether the term significantly improves the model. Where interaction coefficients were significant, we ran post hoc regressions (FDR‐corrected *p*‐values reflect adjustment by the total number of possible relations) to probe subgroups, assessing the source and direction of significant differences.

## 3 RESULTS

### 3.1 Behavioral analyses

Participants’ mean RT (calculated per participant within each sensory modality) was correlated with mean anticipatory alpha‐range LI, *r* = 0.451, *p* < 0.05. Participant's stimulus detection accuracy, consisting of the percent of correct responses (defined as no false alarms or missed hits) over the total number of potential trials (calculated per participant within each sensory modality) was calculated. Participants overall correctly responded to 97.21% of the stimuli. This did not differ between adults and children nor did it correlate with other study measures; thus, we proceeded to focus on RT as the primary indicator of stimulus detection.

Repeated‐measures analyses of variance (ANOVAs) were carried out on RT (from correct trials only) with the following within‐subjects factors: spatial cue direction (right/left), modality (visual/tactile/auditory). Age group (child/adult) was included as a between‐subjects factor. There were no main effects of spatial cue direction. Significant main effects of age group were observed for RT, *F*(1,76) = 0.562, *p* < 0.001, *ɳp* ^2^ = 0.609, with adults responding more rapidly than children. Significant main effects of modality were observed on RT, such that visual stimuli elicited slower RTs relative to tactile, *F*(1,76) = 5.902, *p* < 0.001, *ɳp* ^2^ = 0.547, or auditory stimuli, *F*(1,76) = 4.913, *p* < 0.05, *ɳp* ^2^ = 0.206. RTs for tactile and auditory stimuli did not significantly differ from one another, *F*(1,76) = 4.913, *p* < 0.05, *ɳp* ^2^ = 0.206. No significant interactions were found predicting RT by cue, modality, and group, *F*(1,76) = 1.296, *p* = 0.269.

### 3.2 Anticipatory alpha ERSP analyses

#### 3.2.1 Visual alpha

A repeated‐measures ANOVA was conducted to compare anticipatory visual alpha ERSP in the 500‐ms window prior to the visual target stimulus (Table 2). ERSP at occipital sites was entered as a function of electrode (O1/O2) and cue direction (left/right), submitted as within‐subject variables, as well as age group (child/adult) as a dichotomized between‐subjects variable. There was a significant main effect of age group, *F*(1,79) = 4.03, *SS* = 9.318, *p* = 0.046, *ɳp* ^2^= 0.094, with adults overall exhibiting higher mean ERSP than children. A significant two‐way interaction was observed between cue direction and electrode, *F*(1, 79) = 6.37, *SS* = 10.167, *p* = 0.007, *ɳp* ^2^= 0.204. As expected from prior studies and supported by the ERSP scalp maps (Figure 3), this interaction was driven by more negative ERSP (i.e., greater mu desynchronization) at the contralateral site than at the ipsilateral site. There were no further significant main effects or interactions observed.

**TABLE 2:** Repeated measures ANOVA results for anticipatory alpha ERSP by cue, hemisphere, and age group

Anticipatory mu ERSP	Visual alpha ERSP			Sensorimotor mu ERSP			Auditory tau ERSP		
Predictors	*SS*	*F*‐value	*p*	*SS*	*F*‐value	*p*	*SS*	*F*‐value	*p*
Cue	0.107	0.046	0.830	0.779	0.099	0.830	0.032	0.020	0.887
Hemisphere	4.887	2.113	0.148	5.783	2.113	0.148	0.397	0.367	0.547
Group	9.318	4.030	**0.046**	3.981	2.891	0.126	0.576	0.105	0.748
Cue × Group	4.475	1.935	0.166	4.475	2.444	0.236	0.630	0.582	0.449
Cue × Hemisphere	10.167	6.397	**0.007**	13.167	5.397	**0.009**	0.029	0.060	0.808
Hemisphere × Group	0.764	0.330	0.566	1.840	1.636	0.721	0.059	0.037	0.848
Cue × Hemisphere × Group	0.825	0.357	0.551	17.934	6.247	**0.026**	1.892	3.859	*0.055*

Statistically signfigcant *p* values are denoted by bold.

#### 3.2.2 Sensorimotor mu

A repeated‐measures ANOVA was conducted to compare anticipatory mu ERSP in the 500‐ms window prior to the tactile target stimulus. ERSP at central sites was entered as a function of electrode (C3/C4) and cue direction (left/right), submitted as within‐subject variables, as well as age group (adult/child), which was submitted as a dichotomized between‐subjects variable. No main effects were observed. A significant two‐way interaction was observed between cue direction and electrode, *F*(1, 79) = 5.397, *SS* = 13.176, *p* = 0.009, *ɳp* *^2^* = 0.489. As expected from prior studies and supported by the ERSP scalp maps (Figure 3), this interaction was driven by more negative ERSP (i.e., greater mu desynchronization) at the contralateral site than at the ipsilateral site. A three‐way interaction was observed among electrode site, cue direction, and age group, *F*(2, 76) = 6.247, *SS* = 17.934, *p* = 0.026, *ɳp* *^2^* = 0.264. The interaction appeared to be driven by the greater differences in lateralization of mu ERSP observed in adults: using FDR‐corrected pairwise comparisons, it was determined that the positive ERSP (i.e., an increase in alpha power, or synchronization) observed in adults at ipsilateral central sites (*M* = 0.241) was significantly greater relative to that of children, who exhibited minimal difference in ERSP from baseline (*M* = −0.031).

#### 3.2.3 Auditory tau

A repeated‐measures ANOVA was conducted to examine variability in anticipatory tau rhythm ERSP in the 500‐ms window prior to the target auditory stimulus. ERSP at parietal sites was entered as a function of electrode (P7/P8) and cue direction (left/right), submitted as within‐subject variables, as well as age group (adult/child), which was submitted as a dichotomized between‐subjects variable. No significant main effects or two‐way interactions were observed. A marginally significant, three‐way interaction was observed among electrode site, cue direction, and age group, *F*(2, 76) = 3.859, *SS* = 1.892, *p* = 0.055, *ɳp* *^2^* = 0.129. The interaction appeared to be driven by the greater differences in lateralization of tau ERSP observed in adults: using FDR‐corrected pairwise comparisons, it was determined that the mean ERSP observed in adults at ipsilateral parietal sites (*M* = 0.474) was significantly greater relative to that observed at contralateral parietal sites (*M* = 0.023), while children did not exhibit differences in ERSP at ipsilateral parietal sites (*M* = 0.297) and contralateral parietal sites (*M* = 0.245).

### 3.3 Multilevel analyses: Behavioral outcomes by anticipatory alpha ERSP, modality, and group

To examine differences in how behavioral outcomes were related to anticipatory ERSP by age group and modality, a series of multilevel mixed models were conducted, with variability in outcomes (RT and EF) as a function of LI value (calculated as contralateral alpha‐range ERSP at sensory‐specific sites) and its interactions with modality as a within‐subjects factor and age group as a binary between‐subjects factor. Fixed effects examined this main effect and its moderation by modality and age group (Table 3).

**TABLE 3:** Multilevel mixed effects model of behavioral measures by neural measures

**Model**	**(1)** Within‐subject (WS)			**(2)** Between‐subject (BS)			**(3)** Multilevel model		
**Outcomes**	**Reaction time (RT)**			**Executive function (EF)**			**EF ∼ WS + BS + RT**		
	*R* ^2 ^= 0.303			*R* ^2 ^= 0.123			*R* ^2 ^= 0.219		
	AIC = 638.8			AIC = 641.2					
Predictors	**Beta (SE)**	***t*‐value**	***p***	**Beta (SE)**	***t*‐value**	***p***	**Beta (SE)**	***t*‐value**	***p***
(1) WS LI^a^	−0.0 4 (0.05)	−2.52	**0.031**				−0.23 (0.12)	1.94	0.052
WS ERSP * Trial RT							−0.17 (0.08)	2.13	**0.001**
Intercept	0.55 (0.12)	4.44	**0.001**	0.33 (0.14)	2.42	**0.001**	0.36 (0.13)	2.59	**0.001**
(2) BS LI^b^				−0.67 (0.20)	−3.40	**0.001**	−0.66 (0.20)	−3.28	**0.017**
Group	−1.12 (0.18)	−6.34	**0.001**	−0.09 (0.10)	−0.93	0.351	−0.08 (0.11)	−0.94	0.353
Modality	−0.20 (0.17)	−0.12	0.907	0.01 (0.19)	0.01	0.985	0.003 (0.19)	0.18	0.985
LI × Group	0.14 (0.11)	1.24	0.217	0.12 (0.13)	0.98	0.329	0.26 (0.13)	−2.01	**0.046**
LI × Modality	0.27 (0.13)	1.97	**0.047**	0.36 (0.16)	2.13	**0.028**	0.33 (0.15)	2.07	**0.033**
Group × Modality	0.04 (0.25)	0.158	0.874	−0.01 (0.28)	−0.02	0.979	−0.03 (0.28)	0.11	0.914
LI × Group × Modality	−0.30 (0.16)	−1.88	*0.061*	−0.39 (0.18)	−2.12	**0.034**	−0.38 (0.18)	2.09	**0.026**
**Model** *Ӽ* ^2^ (2) = 0.706, *p* > 0.05; **Significance: *p* > 0.05**									

^a^ In the trial‐level (1) and multilevel model (3), trial‐level WS (within‐subject) LI and reaction time are centered by subject, which ensures its value represents intraindividual variation only. Thus, trial‐level WS LI is not correlated (*r* = 0.19, *p* < 0.10) with subject‐level BS LI. The bolded reported *p*‐values are penalized at the family‐wise error rate.

^b^ In the subject‐level (2) and multilevel model (3), subject‐level BS (between‐subject) LI is centered by group, which ensures its value represents interindividual variation, controlling for trial‐level LI and reaction time.

The first model estimated within‐subject variability in RT (across trials) using within‐subject LI as a predictor, testing how this varied as a function of its interaction with modality and age group. To account for interindividual differences, LI was centered by a participant, allowing analyses to highlight the association between intraindividual variability in LI and trial‐specific RT. As expected, a main effect of LI on RT was evident, such that an increase by one SD of LI for a given trial conferred a 0.40‐s decrease in RT. A main effect of age group was observed, such that children exhibited more variable (and slower) RT than adults. There was an interaction between group and LI, such that children exhibited a nonsignificant association between RT and LI, while in adults, LI was significantly associated with RT. A trending three‐way interaction indicated that this effect might be further moderated by modality, such that the significant association between RT and adult LI was evident for in anticipation of visual and tactile stimuli, but not auditory stimuli.

**FIGURE 4:**
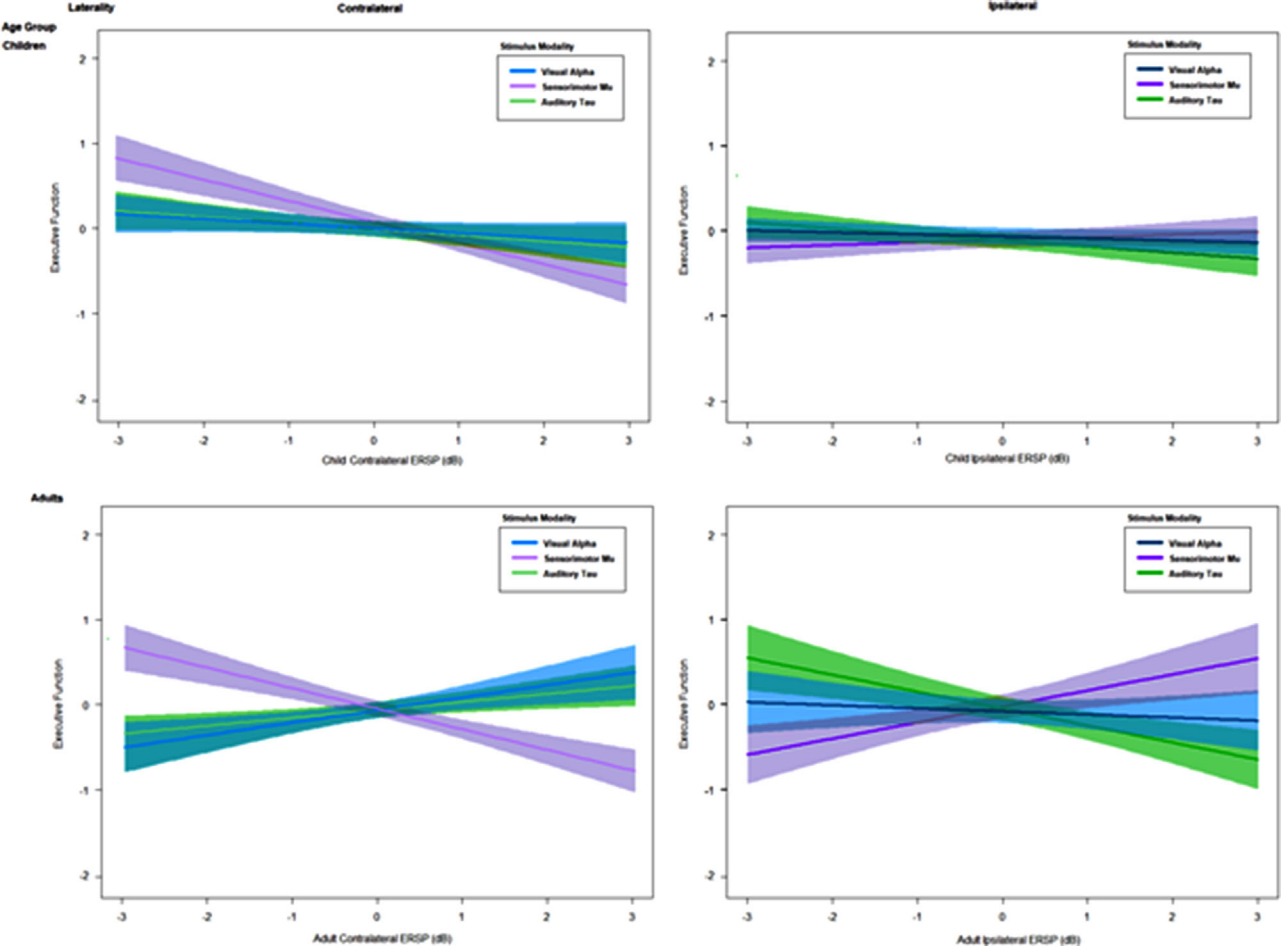
Anticipatory alpha ERSP by sensory modality, presented by age group and lateralization. The interaction of modality and alpha ERSP is displayed, separately (“conditional”) for each age group (child/adult) and laterality, controlling for within‐subject variability in ERSP and its relations to reaction time at the single‐trial level. Contralateral and ipsilateral between‐subject mean ERSP are displayed on the *X*‐axis, with standardized, group‐centered executive function scores displayed on the *Y*‐axis.

The second model estimated between‐subject variability in EF using between‐subject LI as a predictor, examining how this relation varied as a function of LI's interaction with modality and age group. An inverse association of LI with EF was observed, such that participants with lower LI exhibited greater EF scores (evident in 24/40 children and 37/40 adults). There was an interaction between modality and LI in predicting EF variability, such that mu ERSP in anticipation of tactile stimuli was associated with EF, while there was no significant association for alpha LI in anticipation of visual stimuli and tau LI in anticipation of auditory stimuli. This relation was further moderated by age group, yielding a significant three‐way interaction, *t* (78) = −2.12, *p* = 0.034, ɳp*^2^* = 0.092. The regression results are displayed in Figure 4.

To detect the source of significance in the three‐way interaction, a set of post hoc regressions was conducted (with *p*‐values adjusted for multiple comparisons using the FDR correction). EF was defined as a function of anticipatory ERSP values and their interaction with modality, examined separately by age group and ERSP laterality (contralateral/ipsilateral). Contralateral alpha ERSP at occipital sites was not associated with EF scores in children, *t* (39) = −0.05, *β* = −0.01, *p* = 0.96, or adults, *t* (39) = −0.63, *β* = −0.12, *p* = 0.53. Ipsilateral alpha ERSP was not associated with EF abilities in children, *t* (39) = 0.47, *β* = 0.08, *p* = 0.64, or adults, *t* (39) = 1.69, *β* = 0.26, *p* = 0.33. Contralateral tau ERSP at parietal sites was not associated with EF abilities in children, *t* (39) = −0.09, *β* = −0.45, *p* = 0.65, or adults, *t* (39) = −0.61, *β* = −0.14, *p* = 0.55. Ipsilateral tau ERSP was not associated with EF abilities in children, *t* (39) = 0.12, *β* = 0.03, *p* = 0.90, or adults, *t* (39) = 0.82, *β* = 0.17, *p* = 0.41. Contralateral mu ERSP at central sites was inversely associated with EF abilities in children, *t* (39) = −2.18, *β* = −0.48, *p* = 0.03, and adults, *t* (39) = −2.70, *β* = −0.46, *p* = 0.02. Ipsilateral mu ERSP was *not* associated with EF abilities in children, *t* (39) = 0.82, *β* = 0.151, *p* = 0.42, but it was associated with EF in adults, *t* (39) = 2.07, *β* = 0.44, *p* = 0.03.

The multilevel model combined the first model's intraindividual, single‐trial analyses approach with the interindividual differences approach of the second model, to capture greater variability in EF relative to the second model (Δ*R* ^2 ^= 0.096). The multilevel model predicting EF includes a random intercept as well as a random slope. We found that deviance is smaller in the random slope model (*D*_1 _= 2555.1), indicating less model‐data deviance when the effect of within‐subject ERSP varies randomly compared to when only between‐subject ERSP varies (*D*_0 _= 2556.9). The chi‐square value indicates that the random intercept initial model and random slope models are not equal in accounting for variation in EF, *Ӽ* ^2^ (2) = 0.706, *p* > 0.05. The deviance test indicates the significant explanatory power gained by allowing the effect of within‐subject ERSP to vary randomly. By specifying a random slope, heteroscedasticity between participants is accounted for by intraindividual variability in ERSP.

There was a marginal main effect of within‐subject LI on EF, such that an increase by one SD of LI for a given participant conferred a 0.23 decrease in standardized EF score. A main effect was observed of between‐subject LI on EF, such that an increase by one SD of LI for a given participant conferred a 0.66 point decrease in standardized EF score. There was an effect of RT by within‐subject LI on EF, such that an increase by one SD by the correspondence between reaction and within‐subject LI, for a given participant conferred a 0.17 point decrease in standardized EF score. There were no other significant main effects. Importantly, despite accounting for significant within‐subject variance in neural and behavioral responses, the three‐way interaction involving between‐subject LI, modality, and age group remained significant, suggesting that this effect is independent of intraindividual differences.

### 3.4 Exploratory temporal analysis: Microstates across modalities and developmental differences

**FIGURE 5:**
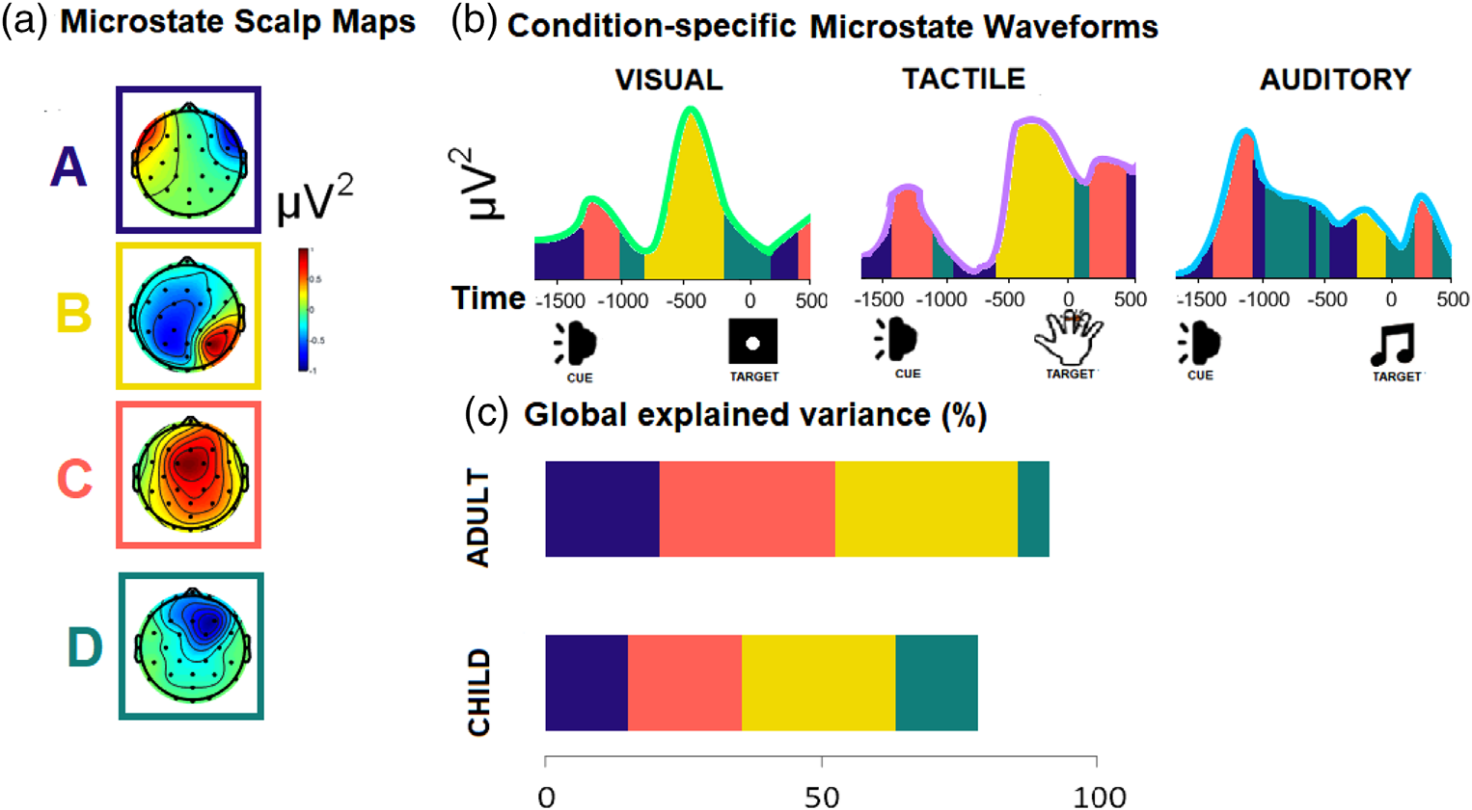
Microstate EEG analysis. (a) Four microstates, labeled A–D, were selected and backpropagated to visualize their sequence in a group‐averaged single trial in each modality condition (b). Together, these microstates explained greater than 70% of the variance in global field power (c).

Using the EEGLAB plug‐in and following the preprocessing steps of Milz et al. (2017), 10 microstate classes were estimated with the existing dataset. The maximum GFP values were identified (Croce et al., 2020), and brief bursty microstates suppressed using a segmentation smoothing algorithm, with a penalty term for nonsmoothness of 0.3 and a window size for smoothing. Microstate functional analysis was applied to samples that were matched across participants in samples per trial and trials per participant, with trials submitted including an identical duration of baseline (−3500 to −1500) and the entire experimental window of analysis (−1500 to 500 following stimulus). Bonferonni‐corrected pairwise *t*‐tests were applied to the fit criteria from resulting clusters, allowing selection of four “prototypical” microstate maps (A–D) that explained a significant proportion of variance. The resulting GFP values and time courses were extracted to and resubmitted in order to perform another *k*‐means clustering (Pascual‐Marqui et al., 1995) searching for four templates to take into account the intersubject variability coming from the previous clustering (Figure 5).

Overall, adults exhibited significantly greater global explained variance (GEV = 0.78) accounted for by functional microstates A–D than children (GEV = 0.71), *t* (76) = 5.61, *p* = 0.021 (Table 4). A linear mixed‐model, which was Bonferroni‐corrected for multiple comparisons on GFP by stimulus and group revealed a significant main effect of stimulus (*χ* ^2^(7) = 15.89, *p*  <  0.001), that varied by both stimulus modality (auditory vs. tactile: *β*  =  0.315, *p*  =  0.009) and also age group (adult vs. child: *β*  =  −0.919, *p*  =  0.001), which was particularly apparent in microstates B (such that the SD of adult GFP was 0.05 uV^2^ lower than children) and C (such that the SD of adults was 0.023 uV^2^ higher in adults). There was also a significant power by age interaction, *χ* ^2^(7)  =  12.51, *p*  =  0.017 with a significant difference between children and adults apparent in microstate B, specific to the alpha range in contrast to the theta range (*β*  =  0.958, *p*  =  0.011) and once again, greater (0.015 uV^2^) in adults than children. In contrast, microstates A, C, and D did not exhibit significant dominance of single power band in determining variability in GFP, although a marginal effect was apparent in contrasting theta and beta power for microstate A (*p* = 0.08).

**TABLE 4:** Percentage of electrode sites per ms with significantly different global field potential in adults and children between EEG microstate classes for the four EEG frequency bands theta (4–7 Hz), alpha (8–12 Hz) and beta (13–30 Hz)

**EEG frequency band**		**A versus B**	**A versus C**	**A versus D**	**B versus C**	**B versus D**	**C versus D**
Theta	Child	0%	5%	2%	8%	1%	2%
	Adult	1%	3%	3%	7%	1%	3%
Alpha	Child	38%	41%	**21%**	**54%**	24%	23%
	Adult	35%	43%	**29%**	**48%**	19%	21%
Beta	Child	1%	3%	2%	5%	2%	4%
	Adult	2%	3%	6%	4%	3%	4%

Bold indicates Bonferroni‐adjusted *p*‐values, equivalent to *p* < 0.0001.

Microstate duration differed only with respect to modality and age–it did not differ as a function of power or the interactions of power by age group or modality. Overall, microstates were briefer in children, lasting 12 ms longer across all microstates in adults and with more frequent bursts or transitions between functional states evident. This was especially apparent in the auditory modality, *β*  = 0.279, *p* = 0.028 compared to the visual modality. Mean duration of microstate B persisted longer in adults (32 ms), *β*  =  0.214, *p*  =  0.031, while microstate D was longer in children (52 ms), *β*  =  0.312, *p*  =  0.019. No significant correlations were evident in relating the GFP or duration of microstates A–D with interindividual variability in RT and EF.

## 4 DISCUSSION

The purpose of the current study was to assess linkages between neural measures of anticipation and variation in behavioral measures of the ability to control action. To accomplish this, we captured trial‐level intraindividual dynamics and subject‐level interindividual variability in anticipatory responses in the EEG signal. We explored variability in alpha‐range oscillatory activity during anticipation of visual, auditory, and tactile stimuli in forty 6– 8‐year‐old children and 40 young adults (undergraduate students aged 18–25 years). The primary findings concern age‐related and modality‐specific effects on changes in EEG alpha power (8–13 Hz) at electrode sites over relevant sensory cortices.

Multilevel analyses followed standard ANOVAs by employing a series of progressive, hierarchical nested models, with the goal of parsing evident intraindividual and interindividual variability in EEG responses. At the trial level, the first model estimated the association (within‐subjects) of RT and an index of the extent of anticipatory lateralization of changes in alpha power, as moderated by modality and participant age group. The multilevel model revealed that individual differences observed in the second, subject‐level model remained significant predictors of EF scores when accounting for within‐subject variability in the extent of anticipatory alpha power lateralization and its association with single‐trial RT.

Consistent with the literature (Chevalier et al., 2013; Fiske & Holmboe, 2019), cue‐facilitated RT was slower in children than in adults, and across both age groups was fastest for tactile and auditory stimuli relative to visual stimuli (supporting hypothesis A). A noteworthy association was found between single‐trial anticipatory changes in visual alpha and RT to visual stimuli: greater anticipatory lateralization of visual alpha was associated with more rapid response times to the target stimulus (partially supporting hypothesis B). This association was apparently driven by adult participants, yet was evident across most participants (including 24 of 40 children). Similar but weaker associations were observed between single‐trial RT to tactile stimuli and the extent of lateralized anticipatory changes in the sensorimotor mu rhythm, with these modest associations evident in both children and adults, confirming prior studies (Meredith et al., 2020). A relation between anticipatory EEG lateralization and RT was not found for auditory stimuli, which is consistent with the mixed literature on anticipatory tau rhythm responses. We speculate that the unimodal nature of the auditory condition (an auditory target following an auditory cue) may have resulted in less salient stimulus anticipation, since participants were not required to shift across modalities (or indeed across spatial locations) between the cue and the target, and thus eliciting less dramatic lateralization differences in anticipation of right and left auditory target stimuli (as noted in hypothesis A and seen in some prior unimodal studies of tau following sequential auditory cues and targets).

EF scores, as the average of age‐standardized, group‐centered scores on Flanker and Card Sort tasks, were significantly associated with individual differences in the extent of anticipatory lateralization of alpha power (supporting hypothesis C). This association was manifested in an interaction between changes in alpha power, sensory modality, and age group. This three‐way interaction was driven by the negative association of scores on the EF tasks with the extent of contralateral alpha desynchronization in children, while for adults, there was a positive association between EF scores and the LI. The subject‐level model accounted for 12% of variability in EF scores; a further 9% of variability in EF scores was accounted for by a multilevel model that incorporated single‐trial variability in alpha power and its interaction with single‐trial RT.

Exploratory functional microstate analyses explored how the temporal dynamics fluctuated across the various events in the task trials by simultaneously considering the signal from electrodes across the scalp to create a global representation of a functional state. Similar to Spadone et al. (2020), we did not find any behavioral indicators to be associated with microstate metrics. Akin to Koenig et al. (2011), we found that the microstates accounted for more than 70% of the variance in task‐evoked EEG spectral modulation of adults and children, with the majority attributed to fluctuations in alpha. Interestingly, comparing the global variance explained and duration as a function of band differences revealed two important developmental differences: first, that microstate B roughly corresponded to the window of analysis for anticipatory alpha fluctuations, and notably accounted for a greater proportion of variance in adults than children. Second, microstate D, roughly corresponding to regulation or transition following peak states B and C, accounted for greater significance and was longer in children, especially when there were only unimodal stimuli presented (auditory spatial cue followed by auditory target stimulus). Microstate A might be attributed as rest or a baseline between other microstates. The similar sequence and duration across trial types (lateralized audiovisual, audiotactile and unimodal auditory) of these microstates might be task‐related. Within our study, microstates complement the multilevel analysis approach by shedding light on the single‐trial dynamics of individual participants; although unlike variability in alpha‐specific power, GFP derived from participant‐averaged microstates across frequency bands (theta, alpha, and beta) did not relate to behavioral measures of EF or RT, they did differ across development.

### 4.1 Specificity of effects and study limitations

Children exhibited minimal changes in anticipatory alpha responses at ipsilateral electrodes over task‐relevant scalp regions, while there was greater variability in changes in alpha power at contralateral electrodes. These results were apparent irrespective of cue direction (left/right). One explanation for these differences in strategic attention allocation between the age groups may be that global inhibition of neural activity (or “rest” itself) is an effortful task for children (Camacho et al., 2020). Thus, children may exhibit more variability in fluctuations of activity at rest, altering the threshold for significant changes such that only contralateral reductions are consistently different across participants. We further highlight developmental differences between children and adults differed by sensory modality, such that adults exhibited greater anticipatory alpha lateralization than children, with this effect driven by heightened alpha synchronization at ipsilateral sites in adults.

In terms of limitations, the cross‐sectional design of the current study constrains directional interpretations of the relations between anticipatory attention and EF. We also note the common role of spatial attention in EF tasks: the ability to anticipate stimulation and the requirement to attend to relevant spatial locations is often integral to EF task demands (Behrmann & Shomstein, 2009; Veer et al., 2017). To partially address this issue, future studies could modify the multimodal selective attention paradigm used in the current study by incorporating simultaneous distractors or by pairing a feature‐based, nonspatial cue with a feature‐based stimulus discrimination task (e.g., Emberson et al., 2015; Kouider et al., 2015).

A relation between EEG activity and RT was not found for auditory stimuli, which is consistent with the mixed literature on anticipatory tau rhythm responses (Frey et al., 2015; Krause et al., 2010). It is speculated that the unimodal nature of the auditory condition (an auditory target following an auditory cue) may have resulted in less salient stimulus anticipation, since participants were not required to shift modalities (or across spatial locations) between the cue and the target. Indeed, sustained attention following the orienting response to the initial cue was evident for a longer duration in both the time‐frequency contrasts and microstate waveforms specifically and only in the unimodal auditory condition. Results indicate the state of stimulus anticipation might be specifically induced by a shift in modality, which in turns elicits greater intraindividual variability in ERSP during this window, and thus ultimately accounts for the gradient in RT apparent within participants (across trials).

### 4.2 Anticipatory attention to the body: Modality‐specific or mu‐specific?

The results of Model 1 suggest that the association of greater anticipatory alpha lateralization with faster RT suggests an age‐independent association between within‐subjects anticipatory alpha power and stimulus processing speed. As evident in the between‐subjects findings of Model 2, the extent of mu desynchronization in anticipation of a tactile stimulus is associated with the ability to control action, as indexed by scores on the EF tasks. Model 3 demonstrated that although the trial‐by‐trial dynamics coupling alpha lateralization and action partially cascade into individual differences in EF scores, meaningful individual differences across the age groups in anticipatory alpha lateralization contribute to variability in EF scores, but only for anticipation of tactile stimulation.

Why might alpha fluctuations in anticipation of tactile stimulation, as opposed to anticipation of auditory or visual stimulation, be related to variability in EF task performance? We suggest the primacy of somatosensation in regulating action, a hypothesis that spans species and evolutionary origins, even to the level of unicellular organisms (Fulkerson, 2014; Lumpkin et al., 2010). It is possible that attention to bodily sensations and variability in perceived boundaries between the body, peripersonal space, and extrapersonal space contributed to the observed developmental and individual differences observed (Bremner et al., 2008). Across development, the processes involved in mu lateralization likely involve spatial attention, action planning, and motor execution, all of which are relevant component processes involved in EF (Aleksandrov & Tugin, 2012; Cannon et al., 2016; Hobson & Bishop, 2017).

Another potential explanation for the mu‐EF relation is that the mu rhythm has a more reliable, lateralized consistency within the individual EEG signal than the auditory tau or visual alpha rhythms. It is possible that the utility of mu as a marker of attentional processes comes in part from this rhythm exhibiting greater interindividual variability and more intraindividual stability than other alpha‐range rhythms (Clayton et al., 2018). The current findings contribute to the developmental cognitive neuroscience literature linking electrophysiological indices of selective attention to cognitive skills in children (Isbell et al., 2016; Shimi et al., 2014) and probabilistic accounts of how prediction develops from infancy through childhood (Baek et al., 2020).

### 4.3 Future directions: Anticipation as a malleable indicator of cognitive development

Neural indices of anticipation can be applied as a tool to investigate the efficacy of selective attention interventions, supporting theories of attention as a flexible, voluntary ability, influenced by instruction, motivation, and environmental demands (Diamond & Ling, 2019; Posner et al., 2012). The findings suggest that attention in the tactile modality might be malleable to improvement, providing a potential a neural account of how interventions can hone bodily attention in adults or improve attentional and executive abilities in young children, including those with developmental differences (Plebanek & Sloutsky, 2017; Stavropoulos & Carver, 2018; Ter Huurne et al., 2017). Situated at the interface of sensory processing and higher‐order cognition, the ability to deploy anticipatory attention is a foundational skill that is present early in life (Johnson et al., 1991; Tarantino et al., 2017; Xie et al., 2018). Our findings suggest important differences between children and adults in the balance of contralateral and ipsilateral anticipatory EEG responses modulate differences in control of action and RT, across sensory modalities. These findings position anticipatory alpha as both responsive to trial‐by‐trial perturbations ahead of action, yet also a consistent measure of interindividual differences in attentional state. As such, anticipatory alpha may be a promising, developmentally consistent neural correlate for measuring the binding of prediction and action (Brown et al., 2011; Feldman & Friston, 2010), useful for testing computational, action‐oriented cognitive neuroscience (Engel et al., 2013; Friston et al., 2011; Huang & Rao, 2011). This study advances an integrative perspective of anticipation while also narrowing down an instance in time and activity in which prediction is evident (Baek et al., 2020; Jaffe‐Dax et al., 2020), with potential for considering how individual differences in behavior and cognitive abilities reflect the accumulation of multilevel, multimodal developmental processes (Marshall, 2014).

## CONFLICT OF INTEREST

The authors declare no competing interests.

## Supporting information

Supplementary information

Supplementary information

## Data Availability

This study was preregistered and uploaded to an OSF Project on 07/16/2018. Also included in the OSF Project is data for adult participants (for whom sharing is permitted; per ethics and recruitment partners, data sharing for child data is not permitted) and R syntax, which can be viewed at the following link: https://osf.io/x2gry/
