# Supplementary material for: Anticipation across modalities in children and adults: Relating anticipatory alpha rhythm lateralization, reaction time, and executive function
Source: Dev Sci. 2022 Jun 7;26(1):e13277. doi: 10.1111/desc.13277 (PMC10078525; doi:10.1111/desc.13277)
Supplement: Supplementary file 1 — Supplementary information [file DESC-26-0-s001.docx]

Supplemental Materials

### Measurement of Executive Function in Childhood

Executive function (EF) batteries in older children typically include assessments spanning multiple neurocognitive capacities, with facets of working memory, cognitive flexibility and inhibitory control (Miyake et al., 2000; Diamond, 2013) differentiated by latent measurement models in children older than ten years of age. Working memory refers to the ability to retain and manipulate information, while cognitive flexibility includes the sustaining or shifting in attention in response to different demands or to apply different rules in different setting, and inhibitory control dictates attentional priorities and restraint of impulsive actions (Miyake & Freidman, 2012). EF task batteries typically index a number of these cognitive processes, particularly in children when they are overlapping, in order to quantify the latent variability shared across EF abilities (Zelazo et al., 2013).

General EF abilities can be indexed by “marker” tasks that involve the coordination of competing attentional priorities. For example, the task demands of the widely used Dimensional Change Card Sort (DCCS) involve all three domains of EF (Frye, Zelazo, & Palfai, 1995; Beck, Schaefer, Pang, & Carlson, 2011; Zelazo, 2006). Directed to match the central test stimuli with one of two lateralized target stimuli by shape or color, child participants on the first block of the task sort the cards by one dimension, and on the next block sort the same cards by the other dimension. Another widely used example is the Flanker task, in which participants indicate the direction of a central arrow that was presented between distractor or ‘flanker’ arrows. The direction of arrows is randomized by trial, such that the flanking arrows are alternatively congruent or incongruent with the target central arrow. Compared with the DCCS, the Flanker is considered a more specific ‘conflict’ task: it primarily indexes inhibitory control (Diamond, 2013), such that incongruent conditions are associated with slower reaction times, which is explained as participants reconciling the conflict between the target and distractor.

The DCCS task indexes task-switching and working memory abilities in childhood (Beck et al., 2011; Zelazo, 2006). Participants were directed to select one of two test stimuli that matched the shape (truck or ball) or color (red or blue) of the target stimuli, as instructed by a verbal prompt that varied randomly between trials. The Flanker task requires participants to indicate the direction of a central arrow that was presented between distractor or ‘flanker’ arrows (Eriksen & Eriksen, 1974; Fan et al., 2002; Rueda et al., 2004). The direction of arrows was randomized by trial, such that the flanking arrows were alternatively congruent or incongruent with the target central arrow. The Flanker task indexes response inhibition and conflict monitoring (Fan et al., 2002; Diamond, 2013). However, the statistical overlap is so high on these tasks that others using identical tasks from the NIH toolbox have also opted to collapse them (Wilboughy et al., 2018; Chen et al., 2019), following the guidelines for the ‘executive function’ domain of the 'Cognitive domain’ for the utility of the NIH toolbox. Our multi-modal selective attention tasks are not refined enough to address the question if the association of alpha lateralization is specific to either inhibitory control (Flanker) or updating/flexibility (Card Sort), as it appears to index the common overlapping individual differences in executive function. A wider range of selective attention tasks accompanied by EEG (i.e. with concurrent presentation of tactile or multi-modal distractors) might make progress identifying a more specific and ‘underlying’ component of executive function.

*Participant Recruitment and Demographic Information*

Ethical approval for the study protocol, consent, assent and questionnaires was approved by the University IRB. Child participants aged between 6 and 8 years were recruited using mailings of a postcard to parents whose addresses were obtained using birth records, flyers posted at businesses frequented by parents and members of the public, and advertisements placed at parent groups, online platforms and social media sites targeting local parents. Families who responded were screened to ensure their children met the age and health criteria; participating families were then emailed a copy of the consent form and provided with a thorough description (via e-mail or over the phone) of the study purpose, protocol, and EEG collection process. Upon entering the lab, parents were briefed on study protocol, requested to consent to the study and then completed surveys in the same room as their participating child, within earshot and available for communication, but out of sight.

Caregivers (on behalf of their participating children) and adult participants completed the MacArthur Sociodemographic Questionnaire, reporting total annual household income within a prescribed set of 10 categories, ranging from under $5,000 to greater than $125,000 (for exact increments, see Kishiyama et al., 2009). Across the entire sample, participants consisted of families from a wide range of income levels, with 16% of participants coming from families with an annual income of under $20,000, and 18% of participating coming from families earning an annual income greater than $125,000. In our child sample, the median income range reported was $50,000 to $74,999; in our adult sample, the median income range reported was $75,000 to $99,999. Highest maternal education attainment was also varied – in the child sample, 6 mothers reported having not attained a high school diploma or equivalent, 12 mothers attained a high school diploma or equivalent, 13 mothers attained an associate or bachelor’s degree, and 9 attained masters, professional or doctoral degrees. In the adult sample, 4 participants reported that their mothers did not attain a high school diploma or equivalent, 14 participants had mothers who had attained a high school diploma or equivalent, 12 participants had mothers with an associate or bachelor’s degree, and 10 had mothers with a masters, professional or doctoral degrees. Participants could report multiple racial identities; if they elected to, they were requested to indicate their primary identity. The ethnic and racial composition of the child sample (ten identified as Hispanic) reflected the demographics of the diverse, multi-cultural metropolitan area that they were recruited from: participating families reported their primary racial identity as White (n = 22), African American (n = 17), Asian (n=3), Native American (n = 1), and Other (n = 4). Adult participants (seven identified as Hispanic) largely reflected the diversity of the undergraduate research participant pool: students reported their as primary racial identity White (n = 21), African American (n = 12), Asian (n = 6) and Other (n = 1).

*Multi-Modal Selective Attention Stimulus Parameters*

Previous studies have established in children and adults that strength of ERSP modulation around a target stimulus is related to cue validity (Voltbregt et al., 2015). Since our goal was to examine individual differences in sensory anticipation, rather than its manipulation under conditions of uncertainty or probabilistic titration, the location of the spatial cue was fully consistent with the subsequent spatial location of target stimuli.

Once consented, participants were fitted with an EEG cap, earphones and tactile stimulators (see below) and were seated at a table facing a computer screen, with instructions to rest their hands on their lap, under the table and out of sight. Whether the foot pedal was assigned to the right or left foot was counterbalanced across participants. Across all blocks, open-field white noise was played in the testing room to facilitate focused attention.

The auditory target stimulus consisted of a high-pitch chime of 200 ms duration, with a 600 Hz initial tone (lasting 100 ms) followed by a 450 Hz offset tone (lasting 100 ms), calibrated to 75 dB SPL at the ear. Visual target stimuli consisted of a white square (2cm x 2cm on the monitor), displayed for 200 ms at a location 4 cm to the right or left of the fixation cross. Tactile stimuli consisted of a light tap, delivered to the distal tip of the left and right middle fingers using an inflatable membrane (10 mm diameter; MEG Services International, British Columbia, CA) mounted in a plastic casing and secured with a finger clip. The membrane was inflated by a short burst of compressed air delivered via flexible polyurethane tubing (3 m length, 3.2 mm outer diameter). The compressed air delivery was controlled by STIM stimulus presentation software in combination with a pneumatic stimulator unit (both from James Long Company, Caroga Lake, NY) and an adjustable regulator that restricted the airflow to 60 psi. To generate each tactile stimulus, the STIM software delivered a 10 ms trigger that opened and closed a solenoid in the pneumatic stimulator. Expansion of the membrane began 15 ms after trigger onset and peaked 35 ms later, with a total duration of membrane movement of around 200 ms. Previous studies have employed this method of tactile stimulation with infants (Shen, Weiss, Metlzoff & Marshall, 2018), children (Weiss, Marshall, & Meltzoff, 2018) and adults (Smyk, Weiss & Marshall, 2018).

The EEG hardware details are available in the supplement methods section. was amplified by optically isolated, high input impedance (>1 GΩ) bioamplifiers from SA Instrumentation (San Diego, CA) and were digitized using a 16-bit A/D converter. Scalp electrode impedances were kept under 25 kΩ. Bioamplifier gain was 4000 for the EEG channels with hardware filter settings of 0.1 Hz (high-pass) and 100 Hz (low-pass), with a 12 dB/octave rolloff. The EEG signals were collected referenced to Cz with an AFz ground, and were re-referenced offline to the average of the left and right mastoids. If visual inspection of the raw EEG signal identified of up to three non-neighboring electrodes with noise exceeding a standard threshold of 100 µV, these were interpolated using EEGLAB’s spherical interpolation function. Movement and eye blink artifacts were detected and interpolated using independent component analysis (Hoffmann & Falkenstein, 2008). The EEG signal was then spectrally analyzed with Gaussian tapered complex Morlet wavelets. Event-related spectral perturbation (ERSP) was computed for frequencies ranging from 4-30 Hz (Jantzen et al., 2012), with analyses focused on the alpha (8-12 Hz) range of oscillatory activity (Delorme, 2004).
