# Supplementary material for: Anticipation across modalities in children and adults: Relating anticipatory alpha rhythm lateralization, reaction time, and executive function
Source: Dev Sci. 2022 Jun 7;26(1):e13277. doi: 10.1111/desc.13277 (PMC10078525; doi:10.1111/desc.13277)
Supplement: Supplementary file 2 — Supplementary information [file DESC-26-0-s002.docx]

**AsPredicted registration:**

###### 1. Have any data been collected for this study already? optional

- Yes, at least some data have been collected for this study already

- **No, no data have been collected for this study yet**

###### 2. What's the main question being asked or hypothesis being tested in this study?

##### A) Does the extent of anticipatory alpha-range EEG modulation differ by sensory modality?

A1. Test for the presence of lateralized tau (auditory), mu (somatosensory) and alpha (visual) anticipatory ERSP by comparing contralateral and ipsilateral power in electrode sites over relevant sensory cortices, 1000 ms before delivery of target stimulus (but after presentation of spatial cue) in different modalities.

A2. Analyses will directly compare modulation between child and adult participants. Unless otherwise noted, all analyses will be conducted and reported separately for adult and child samples.

##### B) Is the extent of anticipatory alpha-range modulation related to behavioral indicators of attention or of stimulus perception?

B1. Reaction time in response to a target stimulus and accuracy of target stimulus detection is considered a useful indicator of attention and perception across trials.

B2. Analyses will first be conducted to test the relation of reaction time and accuracy of detection to mean anticipatory tau/mu/alpha modulation within each modality.

B3. We will then collapse across modalities for each participant and examine anticipatory tau/mu/alpha modulation as a supramodal indicator of anticipation in relation to these behavioral indices of attention/perception.

##### C) Are executive function (EF) abilities related to the extent of alpha-range EEG modulation in anticipation of auditory, tactile or visual stimuli?

C1. Analyses will probe differences in the relations between EF and anticipation across sensory modalities

C2. Analyses will directly compare these relations between child and adult participants.

C3. Contingent upon the results of prior analyses, we will then control for reaction time (B1) and target detection accuracy (B2) to assess whether behavioral indices of stimulus perception influence these relations.

###### 3. Describe the key dependent variable(s) specifying how they will be measured. (optional)

##### A) Participants

Adult and child participants (N=100) will be recruited, in order to retain a sample size of n=40 in each the adult (18+) and child (6-8) age range which is powered sufficiently (.80) to detect our expected medium effect size (η2p = 0.09), given the number of parameters and multi-level regression analyses to be conducted. Participants were required to have no medical or psychological diagnoses, be right-handed, and be free of long-term medication. Adult undergraduate participants (n=50) will be recruited using Temple University’s SONA system, which awards course credit to students. Based on prior experience with a similar paradigm, we can expect to recruit 60-65 children in order to achieve a final n=50, with participant loss due to reasons including excessive artifact, intolerance to EEG cap, technical issues with recording and ineligible participants. Child participants ages 6-8 years will be recruited using mass mailings of a flyer explaining the study and soliciting families to call if they would like to participate. The mailings are sent to families’ homes, with the addresses and child ages received from the Bureau of Health Statistics & Registries of the Pennsylvania Department of Health through their “Vital Statistics” records (the PI has approval to do this). Families are mailed a flyer explaining our laboratory’s research. If families are interested upon reading the flyer, they call or email our study coordinator. If the children meet the age and health criteria, the study coordinator will outline and discuss the details of the study and EEG collection with the parent/guardian. If the parent or guardian expresses interest in participating, the study coordinator schedules them for a visit to the PI’s laboratory in Weiss Hall on main campus. Parents will be compensated with a $50 Visa gift card for their participation, and children will receive a small toy and certificate of participation.

##### B) Selective Attention Protocol

Prior to data collection, adult participants will be consented and child participants will be read an assent form in the presence of their caregiver that outlines the experimental protocol. Participants will then be fitted with an EEG cap, earphones and tactile stimulators (see below) and were seated at a table facing a computer screen, with instructions to rest their hands on their lap, under the table and out of sight.

The multi-sensory selective attention paradigm (Figure 1) will consists of three blocks - auditory, tactile and visual - indicated by a visual icon (an ear, a hand or an eye) displayed at the start of each block, and experimenters will instruct participants that an arrow cue will prepare them for stimulation delivered in the specified sensory modality. Order of blocks (modality) presented will be counterbalanced to six possible protocol sequences, which will be randomly assigned across participants. At the start of each block, there will be 6 practice trials in the attended modality, such that participants will have experienced completed a trial of each spatial manipulation and successfully discriminated between single and double stimuli (explained below). Participants will then be told to minimize movement and propped into a chin rest, adjusted to a comfortable height.

The selective attention protocol for each trial involves a baseline fixation cross (+) displayed on a CRT monitor for 500 ms. The baseline is followed by an auditory spatial cue a presented to either headphone for 200 ms that directs participants attention to the right or left or both (control), with the goal of eliciting anticipation of upcoming sensory stimuli. Target sensory stimuli will be presented 1500 ms after the cue onset, consisting of an auditory tone to the cued ear (distinct from the cue), tactile pulses of target stimuli to the cued hand, or visual probes in the cued field of vision. At the onset of the target stimulus (0 ms) the directional arrow will remain displayed for an additional 500 ms (even for blocks with visual targets). Following 500 ms after the presentation of the target stimuli trial, the screen will display an exclamation point (!) for 2000 ms, which instructs participants to report the number of stimuli detected by pressing a foot pedal. An inter-trial interval of 500 ms will buffer each trial, for a total trial duration of 5 seconds.

There will be 430 trials: 120 trials of each modality (presented in separate blocks, with opportunity for rest in between) and 60 to each spatial location (← or→ ) and 30 control (with auditory stimuli presented to both ears simultaneously) within each modality (auditory, tactile or visual). Among these, 20 randomly-interwoven trials will consist of ‘double’ stimuli, in which an additional stimulus in the attended modality will be presented to the cued spatial location; participants will be instructed to respond to these trials with two rapid foot pedal presses during the response window (!). Detection of these double stimuli (along with false alarms) will form an accuracy score, which will serve as a behavioral indicator of participants’ attention. In addition to calculating participant accuracy as a percent of total artifact-free EEG trials (within each sensory modality), behavioral indicators of task performance will be assessed with variability in reaction time, collected via timestamps from response window (!) onset to foot pedal response.

##### B2) EEG Measure of Anticipation: Alpha Modulation

The frequency range of the mu rhythm moves from around 6-9 Hz in infancy to a higher frequency range in early childhood (Berchicci et al., 2011; Marshall et al., 2002). By the age of the current sample (6-8 years) the mu frequency band is close to the adult range of 8-12 Hz (Berchicci et al., 2011) so this frequency range will be used for analyses. For each single-pulse trial with a correct behavioral response, an epoch of 2750 ms will be extracted beginning 2000 ms prior to onset of the tactile stimulus and extending 750 ms after target stimulus onset, prior to the behavioral response window. Initially, a mass univariate approach will be implemented in EEGlab, with pairwise t-tests conducted on the following factors to predict ERSP amplitude across all 32 sampled channels, correcting for multiple comparisons: spatial direction of cue (right/left), sensory modality (auditory/tactile/visual) and participant age (adult/child).

Using the results of the mass univariate scalp maps as a guide, a key variable submitted to ANOVAs and regressions will be mean mu ERSP for the period from -500 ms to 0 ms*, with this time window selected to prevent contamination of anticipatory responses by changes evoked by the visuospatial cue that occurred at -1500 ms. Given that there are no significant differences by directional cue, mean ERSP will be collapsed into contralateral ERSP and ipsilateral ERSP. This plan for analyses is based on prior work on child and adult anticipatory mu modulation and is similar to the parameters (duration of anticipatory period and alpha-range criteria) used by the study of cross-modal visual-auditory study of children (8-12) and adults (Murphy, Molholm, and Foxe, 2016), but we acknowledge that adjustments may occur based on visual inspection of the group-level ERSP waveforms and scalp maps.

##### B3) Behavioral Measure of Post-Stimulus Perception/Action Execution

Participants’ mean reaction time, consisting of time from initial response screen onset (750 ms) to foot pedal press (calculated per participant within each sensory modality) will be correlated with mean anticipatory alpha-range EEG modulation. Participant’s stimuli detection accuracy, consisting of the percent of correct responses (defined as no false alarms or missed hits) over the total number of potential trials (calculated per participant within each sensory modality) will be correlated with mean anticipatory alpha-range EEG modulation.

##### C) Cognitive Skills Battery

Participants will be detached from the EEG and have their executive function abilities assessed using the standardized measures provided in the NIH Cognitive Assessment toolbox. This computerized assessment includes a battery of tests to parse the components of executive function, including cognitive flexibility (Dimensional Change Card Sort) and inhibitory control (Flanker). The dimensional change Card Sort task indexes task-switching and working memory abilities in childhood (Beck et al., 2011; Zelazo, 2006). Participants are directed to select one of two test stimuli which matched the shape (truck or ball) or color (red or blue) of the target stimuli, as instructed by a verbal prompt which varied randomly between trials. The Flanker task is a child-friendly adaptation of the Attention Network Test (Rueda et al., 2004) in which participants are required to indicate the direction of a central arrow that was presented between distractor or ‘flanker’ arrows. The direction of arrows was randomized by trial, such that the flanking arrows are alternatively congruent or incongruent with the target central arrow. The Flanker task indexes response inhibition and conflict monitoring (Diamond, 2013). Participant’s scores on the Card Sort and Flanker tasks are calculated to reflect both accuracy and reaction time for participants who correctly identified targets on 80% of trials; accuracy alone is considered for participants who did not meet this threshold. For all measures on the Cognitive battery, we will use t-standardized test scores (on a scale from 0 to 100, with a mean of 50) which combine the Card Sort and Flanker to provide an index of executive function, as advised by the NIH Cognitive Toolbox, which are adjusted for age and gender.

###### 4. How many and which conditions will participants be assigned to? (optional)

· All participants will undergo all conditions (auditory/visual/tactile), with the order of target sensory modality counterbalanced across participants.

5. Specify exactly which analyses you will conduct to examine the main question/hypothesis. (optional)

For all ANOVAs, within-subject effects will be adjusted using Greenhouse-Geisser correction factors; pairwise t-test comparisons and multiple linear regressions will be reported with p-values adjusted for multiple comparisons using the FDR correction. Three 3-way ANOVAs will be conducted separately to investigate mean ERSP amplitude for each sensory modality during the anticipatory period will be tested for significant differences in hemisphere (contralateral / ipsilateral) and age (child / adult).

Bivariate correlations will investigate how participant stimuli detection accuracy and mean reaction time in response to stimuli relate to participants’ mean anticipatory EEG responses (both calculated within each modality and age range separately).

Regressions will investigate how two aspects of EF (measured individually) are associated with pre-stimulus EEG amplitude or frequency, moderated by cued sensory modality (auditory, visual or tactile), ipsilateral or contralateral hemisphere to cue (right or left), and participant age (child or adult). Regression analyses (conducted within each age range and sensory modality) will involve the executive function score(s) regressed over anticipatory EEG in pre-specified, regionally-specific electrodes and stimuli detection accuracy and reaction time submitted to the first step of a multiple linear regression; on the second step, executive function score(s) will regressed over anticipatory EEG alone, in order to parcel out the variance in executive function captured by behavioral indices and elucidate whether anticipation itself captures additional, unique variance.

6. Any secondary analyses? (optional)

· Additional analyses are planned utilize time series autoregression to identify when the transition from cue response to anticipatory activity is elicited. We will compare the transition from auditory cue to tactile or visual targets with the auditory target block in order to examine if cross-modal vs. uni-modal attention operates differently at the neural level. Further analyses can examine correlations between reaction time and detection accuracy with alpha modulation at the single trial level to identify whether fluctuations of attention occur within participants, and whether some measure of this (to be developed) can be utilized to predict failure of attention.

7. How many observations will be collected or what will determine the sample size? No need to justify decision, but be precise about exactly how the number will be determined. (optional)

· To detect small to moderate effects and study individual differences we need a minimum sample size of 50, and thus will recruit between 60 participants (of children 6-8 and adults) expecting to discard (particularly in the child sample) roughly 10-15% of recruited participants, in anticipation of the following issues 1) ineligibility/intolerance of cap preparation 2) technical issues precluding collection of EEG 3) less than 25 trials / condition (6 usable conditions)

8. Anything else you would like to pre-register? (e.g., data exclusions, variables collected for exploratory purposes, unusual analyses planned?) (optional)

Secondary analyses will be conducted on the correlation between mean alpha modulation (in all 3 modalities separately and collapsed across modalities) with participant’s self-reported use of technology, which is measured using the smartphone addiction scale, problematic phone use, self-reported hours of technology use, and social media engagement survey. The goal of these exploratory analyses is to assess whether patterns of mobile phone and social media usage are associated with individual differences in neural indicators of attention.
